# The FSHD muscle–blood biomarker: a circulating transcriptomic biomarker for clinical severity in facioscapulohumeral muscular dystrophy

**DOI:** 10.1093/braincomms/fcad221

**Published:** 2023-08-16

**Authors:** Christopher R S Banerji, Anna Greco, Leo A B Joosten, Baziel G M van Engelen, Peter S Zammit

**Affiliations:** Randall Centre for Cell and Molecular Biophysics, King’s College London, London SE1 1UL, UK; The Alan Turing Institute, The British Library, London NW1 2DB, UK; Department of Neurology, Donders Institute for Brain, Cognition and Behaviour, Radboud University Medical Center, 6525 GA Nijmegen, The Netherlands; Department of Internal Medicine, Radboud Institute of Molecular Life Sciences (RIMLS) and Radboud Center of Infectious Diseases (RCI), Radboud University Medical Center, Nijmegen 6525, The Netherlands; Department of Internal Medicine, Radboud Institute of Molecular Life Sciences (RIMLS) and Radboud Center of Infectious Diseases (RCI), Radboud University Medical Center, Nijmegen 6525, The Netherlands; Department of Medical Genetics, Iuliu Hatieganu University of Medicine and Pharmacy, 400012 Cluj-Napoca, Romania; Department of Neurology, Donders Institute for Brain, Cognition and Behaviour, Radboud University Medical Center, 6525 GA Nijmegen, The Netherlands; Randall Centre for Cell and Molecular Biophysics, King’s College London, London SE1 1UL, UK

**Keywords:** facioscapulohumeral muscular dystrophy, FSHD, circulating biomarker, DUX4, PAX7

## Abstract

Facioscapulohumeral muscular dystrophy (FSHD) is a prevalent, incurable skeletal myopathy. Clinical trials for FSHD are hindered by heterogeneous biomarkers poorly associated with clinical severity, requiring invasive muscle biopsy. Macroscopically, FSHD presents with slow fatty replacement of muscle, rapidly accelerated by inflammation. Mis-expression of the transcription factor DUX4 is currently accepted to underlie pathogenesis, and mechanisms including PAX7 target gene repression have been proposed. Here, we performed RNA-sequencing on MRI-guided inflamed and isogenic non-inflamed muscle biopsies from the same clinically characterized FSHD patients (*n* = 24), alongside isogenic peripheral blood mononucleated cells from a subset of patients (*n* = 13) and unaffected controls (*n* = 11). Multivariate models were employed to evaluate the clinical associations of five published FSHD transcriptomic biomarkers. We demonstrated that PAX7 target gene repression can discriminate control, inflamed and non-inflamed FSHD muscle independently of age and sex (*P* < 0.013), while the discriminatory power of DUX4 target genes was limited to distinguishing FSHD muscle from control. Importantly, the level of PAX7 target gene repression in non-inflamed muscle associated with clinical assessments of FSHD severity (*P* = 0.04). DUX4 target gene biomarkers in FSHD muscle showed associations with lower limb fat fraction and D4Z4 array length but not clinical assessment. Lastly, PAX7 target gene repression in FSHD muscle correlated with the level in isogenic peripheral blood mononucleated cells (*P* = 0.002). A refined PAX7 target gene biomarker comprising 143/601 PAX7 target genes computed in peripheral blood (the FSHD muscle–blood biomarker) associated with clinical severity in FSHD patients (*P* < 0.036). Our new circulating biomarker validates as a classifier of clinical severity in an independent data set of 54 FSHD patient and 29 matched control blood samples, with improved power in older patients (*P* = 0.03). In summary, we present the minimally invasive FSHD muscle–blood biomarker of FSHD clinical severity valid in patient muscle and blood, of potential use in routine disease monitoring and clinical trials.

## Teaser

Lima-Filho, Benedet *et al*. reported that cognitively unimpaired elders carrying the *FNDC5* allele rs1746661(T) develop brain low glucose metabolism and increased amyloid deposition. These findings indicate that *FNDC5* may contribute to regional glucose metabolism in the human brain.

## Graphical Abstract

**Graphical Abstract:**
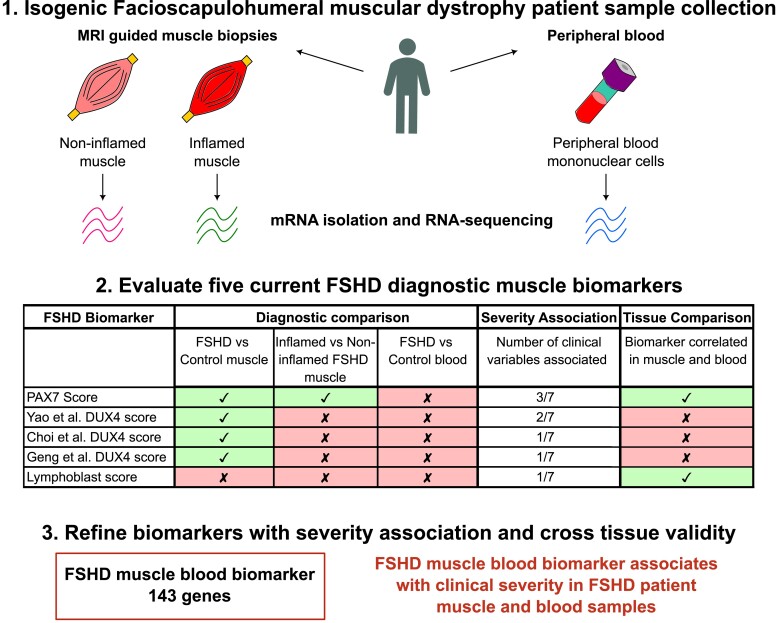

See Kyba and Bosnakovski (https://doi.org/10.1093/braincomms/fcad235) for a scientific commentary on this article.

## Introduction

Facioscapulohumeral muscular dystrophy (FSHD) is a prevalent (∼12/100 000^1^), incurable, inherited myopathy. FSHD is slowly progressive and highly heterogeneous both clinically and molecularly,^2^ making reliable biomarkers for clinical severity and progression challenging to develop. However, such biomarkers are needed for monitoring FSHD patients and measuring outcomes in clinical trials. Moreover, gene-based biomarkers facilitate understanding of pathomechanisms and inform therapeutic development.

FSHD is linked to epigenetic derepression of the D4Z4 macrosatellite at chromosome 4q35 alongside a permissive 4qA haplotype.^3,4^ Epigenetic derepression is achieved by two mechanisms.^5^ The most common (95% of patients, FSHD1, OMIM: 158900) involves truncation of the D4Z4 region from the typical >100 repeats to 10-1 units.^6,7^ Mutations in epigenetic modifiers, typically *SMCHD1*^8^ (or rarely *DNMT3B*^9^ or *LRIF1*^10^), drive epigenetic derepression in the remaining 5% (FSHD2, OMIM: 158901). Each D4Z4 repeat encodes a pioneer transcription factor termed DUX4,^11,12^ and epigenetic derepression permits transcription of DUX4 from the distal-most D4Z4 unit.^13,14^ In both FSHD1 and FSHD2, DUX4 transcripts are stabilized by splicing to a poly(A) signal in the flanking DNA on permissive 4qA haplotypes, permitting translation.^4^

DUX4 is expressed during zygotic genome activation, before being epigenetically repressed in somatic tissues.^15^ Mis-expression of DUX4 is believed to underlie FSHD pathogenesis but is extremely difficult to detect in FSHD muscle, with protein found in ∼1/1000 FSHD myoblasts *ex vivo*.^2,16^ Despite this, DUX4 target gene biomarkers associate with FSHD status, particularly in the context of active inflammation (e.g. STIR/TIRM positivity on MRI).^17-22^

The two DNA-binding homeodomains of DUX4 show homology to the single homeodomain of the myogenic master regulator PAX7,^23^ suggesting overlap in DUX4 and PAX7 function.^18,24^ We have demonstrated that PAX7 target gene repression hallmarks FSHD muscle regardless of inflammatory state, associating with histological and MRI measures of pathological severity.^17-19^ Our PAX7 target gene repression biomarker has been independently verified.^20,22^ Importantly, PAX7 target gene repression progresses in muscle biopsies from the same FSHD patient 1 year apart, associating with disease duration.^25^

Therapeutics for suppressing DUX4 have reached clinical trials, including the p38 inhibitor losmapimod.^26^ However, losmapimod did not significantly change the primary outcome measure: DUX4 target gene expression in FSHD muscle, despite improvement in secondary functional outcomes. Though this may be attributable to several factors, including losmapimod also acting on unrelated pathways, it motivates development of more sensitive biomarkers of FSHD severity for monitoring clinical trials. Moreover, biomarkers based on muscle biopsies are invasive, and FSHD muscle may not mount an appropriate repair/regenerative response,^27^ necessitating minimally invasive measures.

Circulating biomarkers for FSHD have been investigated via discovery approaches. Proteomics of muscle microdialysates identified innate immunity mediators S100-A8 and A9^28^ as FSHD associated. Studies of FSHD plasma also found S100-A8, several miRNAs^29^ and complement components.^30^ We defined a set of genes over-expressed in FSHD lymphoblastoid cell lines, which correlate with immune infiltration in FSHD muscle biopsies.^19^ Recently, a large RNA-sequencing study on peripheral blood found no transcripts differentially expressed between FSHD patients and controls or associated with FSHD clinical severity.^31^ Conversely, serological studies identified IL-6,^32^ TNF,^33^ and structural muscle components^34^ as associated with FSHD severity: how the circulating levels of these factors relate to FSHD muscle tissue is unknown.

FSHD is a muscle pathology, accelerated by inflammation,^2^ and informative blood-borne biomarkers should correlate directly to muscle-based markers of severity. To identify biomarkers of clinical severity valid in both muscle and blood, we performed RNA-sequencing on 24 clinically characterized FSHD patients, obtaining isogenic non-inflamed (TIRM^−^) and inflamed (TIRM^+^) muscle biopsies and peripheral blood mononucleated cells (PBMCs), alongside muscle and PBMCs from control subjects. We computed DUX4 and PAX7 biomarkers and identified associations between biomarker expression levels and measures of clinical severity. There was clear association between levels of PAX7 target gene repression in muscle and blood but not DUX4 target gene expression. Importantly, a refined 143 PAX7 target gene signature (termed the ‘FSHD muscle–blood biomarker’) in PBMCs correlated with widely used FSHD clinical severity scores defined in Ricci *et al*.^35^ and Lamperti *et al*.^36^ Investigation of our FSHD muscle–blood biomarker on an independent data set of 54 FSHD and 29 matched control blood samples validated it as a minimally invasive biomarker of FSHD severity, with particular relevance to older patients.

## Materials and methods

### Participants

The study was approved by the regional medical ethical committee approval (CMO Arnhem-Nijmegen) under file number NL64690.091.18. Subjects provided written informed consent. FSHD patients (*n* = 25 FSHD1 and 1 FSHD2) and unrelated healthy control individuals (*n* = 35) were recruited from 2019 to 2021 at the neurology outpatient clinic, Radboud University Medical Center, Nijmegen, The Netherlands. Patient criteria were as follows: (i) genetically confirmed FSHD, (ii) age ≥ 18 years, (iii) absence of infectious and/or inflammatory comorbidities even if transient, (iv) absence of autoimmune comorbidities, (v) absence of past/present malignancy, and (vi) no use of corticosteroids, statins or anti-inflammatory medication. Unrelated healthy control individuals had to fulfil criteria (ii) to (vi) from the patient group, in addition to the following: (i) negative personal/family medical history for neuromuscular disorders and (ii) absence of muscle weakness during physical examination.

Clinical assessments Ricci clinical severity score^35^ (Ricci), Lamperti clinical severity score^36^ (Lamperti), Medical Research Council (MRC) sum score, maximum voluntary contraction (MVC) of tibialis anterior (TA), disease duration and D4Z4 array length, PBMC acquisition, muscle MRI [including assessment of TIRM status and lower limb fat fraction (LLFF)] and MRI-guided muscle biopsy were performed following standard procedures detailed in Supplementary Methods.

All 26 FSHD patients underwent clinical assessment. A total of 24/26 FSHD patients underwent two muscle biopsies each, one from a non-inflamed muscle (TIRM^−^ on MRI) and one from an inflamed muscle (TIRM^+^ on MRI), resulting in 24 TIRM^−^ samples and 24 corresponding isogenic TIRM^+^ samples. Fifteen FSHD patients were sampled for PBMCs, of these 13/15 also underwent muscle biopsies and so had corresponding isogenic TIRM^−^ and TIRM^+^ samples, while 2/15 had no corresponding muscle biopsy samples (Supplementary Fig. 1).

All 23 control individuals underwent clinical examination. Eleven control individuals underwent muscle biopsy of the vastus lateralis. Fourteen control individuals were sampled for PBMCs, and 2/14 control individuals sampled for PBMCs also underwent muscle biopsy (Supplementary Fig. 1).

### Analysis of clinical variables

Clinical variables assessed in all participants included age at examination, sex and MRC muscle power grade. Variables were compared between FSHD and control individuals in three settings: entire cohort, subset of cohort in which muscle biopsies were performed and subset of cohort in which PBMCs were isolated. Age at examination and MRC sum score were compared in these three settings via Wilcoxon test, while sex was compared via logit regression, and significance was assessed at *P* < 0.05.

FSHD severity indicators, Ricci, Lamperti and MRC sum score, MVC of TA, LLFF, D4Z4 array length (FSHD1 patients) and disease duration, were compared across the 26 FSHD patients in the full cohort. Pearson’s correlation analysis was performed for each pairwise combination of the seven variables (21 comparisons), *P*-values were adjusted for multiple testing via the Benjamini–Hochberg method and significance was assessed at adjusted *P* < 0.05.

### RNA-sequencing

RNA extraction from muscle biopsies, purification, quality control, library preparation and sequencing at 21.7–35.5 million reads/sample was performed by Genewiz (https://www.genewiz.com). RNA was extracted from PBMCs followed by globin depletion, quality control, library preparation and sequencing at 19.7–46.5 million reads/sample performed by Genewiz (https://www.genewiz.com). Further quality control, alignment and mapping were performed following standard procedures summarized in Supplementary Methods.

### Computation and diagnostic assessment of FSHD biomarkers

Computation of three DUX4 target gene expression biomarkers (Choi *et al*.,^37^ Geng *et al*.^38^ and Yao *et al*.^39^), PAX7 target gene repression biomarker and Lymphoblast score were as previously described.^17-19,25^ Biomarker values in control samples (14 PBMCs and 11 muscle biopsies) were compared with corresponding FSHD samples (15 PBMCs, 24 TIRM^−^ and 24 TIRM^+^ muscle biopsies), via multivariate linear regression and the ‘globaltest’ Bioconductor package^40^ adjusting for age and sex. Biomarker values in isogenic TIRM^−^ and TIRM^+^ samples were compared via a paired analysis. Significance was assessed at *P* < 0.05, and full details are provided (Supplementary Methods).

### Association between FSHD biomarkers and clinical severity variables and across tissues

For each of the five FSHD biomarkers, Pearson’s correlation analysis was performed separately in 24 TIRM^−^ and 24 TIRM^+^ FSHD muscle biopsies to determine association between each of the seven FSHD clinical severity indicators: Ricci, Lamperti and MRC sum scores, MVC of TA, LLFF, D4Z4 array length (FSHD1 samples only) and disease duration (14 comparisons per biomarker).

For each of the five biomarkers, Pearson’s correlation was also performed to determine association between biomarker levels in 13 TIRM^−^ and TIRM^+^ FSHD muscle samples and corresponding levels in 13 isogenic PBMCS. Significance was assessed at *P* < 0.05.

### Constructing refined target gene signatures

For each of the 601 genes comprising the full PAX7 target gene biomarker (311 upregulated; 290 downregulated),^18^ a Pearson’s correlation analysis was performed investigating association between expression of each gene in the 24 TIRM^−^ and 24 TIRM^+^ FSHD samples separately, and three clinical variables found to associate with the full PAX7 biomarker in muscle (Ricci and Lamperti severity scores and disease duration). We found 143/601 genes (64/311 upregulated; 79/290 downregulated) significantly correlated with one of the three clinical variables across either TIRM^−^ or TIRM^+^ samples at *P* < 0.05.

These 143 genes were employed to construct the ‘FSHD muscle–blood biomarker’. Replicating the construction of the PAX7 target gene repression biomarker, the FSHD muscle–blood biomarker is computed in a single sample as the *t*-statistic evaluating the difference between expression of 64 upregulated PAX7 target genes and 79 downregulated PAX7 target genes.

For each of the 237 genes comprising the full Lymphoblast score,^19^ a Pearson’s correlation analysis was performed investigating association between expression of each gene in the 24 TIRM^−^ and 24 TIRM^+^ FSHD samples separately and LLFF—found to associate with the full Lymphoblast score in muscle. We found 44/237 genes significantly correlated with LLFF across either TIRM^−^ or TIRM^+^ samples at *P* < 0.05. The mean expression of these 44 genes constituted the Refined Lymphoblast score.

Multivariate linear regression adjusting for age and sex determined association between the refined scores in 15 FSHD PBMCs and clinical variables: Ricci, Lamperti scores and disease duration for the refined PAX7 score and LLFF for the Refined Lymphoblast score at the 5% significance level.

### Validation of the FSHD muscle–blood biomarker

The 143-gene FSHD muscle–blood biomarker was computed in the 11 controls, 24 TIRM^−^ and isogenic TIRM^+^ muscle biopsies and 15 FSHD PBMCs. Comparison between control and FSHD samples was as described for the full PAX7 score. Pearson’s correlation analysis determined association between the FSHD muscle–blood biomarker in 13 TIRM^−^ FSHD muscle biopsies and 13 isogenic PBMCs.

The FSHD muscle–blood biomarker was also computed on 26 isogenic Year 1 and Year 2 muscle biopsy samples described by Wong *et al*.,^22^ and normalized read counts were obtained from the GEO database accession: GSE115650. Biomarker levels between Year 1 and Year 2 samples were compared via a two-tailed paired Wilcoxon test.

For meta-analysis of the FSHD muscle–blood biomarker as a diagnostic measure in muscle biopsies, seven independent data sets containing transcriptomic assessments of FSHD and control muscle biopsies were downloaded as normalized data sets from the GEO database (Supplementary Methods). These seven data sets describe 228 muscle biopsies (130 FSHD and 98 controls). All data were log transformed and quantile normalized within the study for computation of the FSHD muscle–blood biomarker in line with our previous analyses.^17,19,25^ FSHD muscle–blood biomarker differences between FSHD and control samples were evaluated within each study via two-tailed Wilcoxon test and meta-analysis across the seven studies using a random effects model, with overall significance assessed via Fisher’s combined test.

### Analysis of independent data set of Signorelli *et al*.

Raw read counts describing RNA-sequencing of PAXgene blood from 54 FSHD patients and 29 controls were described in Signorelli *et al*.^31^ PAXgene Blood Tubes employ a proprietary reagent for immediate stabilization of intracellular RNA on blood sampling via venepuncture. This contrasts with our approach to assessing peripheral blood transcriptomics, in which blood was sampled into EDTA-coated tubes and PBMCs isolated by centrifugation before RNA extraction. The Signorelli *et al*. data set comprises two independent cohorts processed separately. The Nijmegen cohort comprises 39 FSHD and 11 control samples and was sequenced to an average depth of 17.5 million reads per sample. The Newcastle cohort comprises 15 FSHD and 18 control samples and was sequenced to an average depth of 43.8 million reads per sample. Clinical annotations included age, sex and Lamperti score. Each data set was normalized separately using the ‘DESeq2’ package in R,^41^ and data were then log transformed and quantile normalized within each cohort in line with our prior analysis.^18,19,25^ The FSHD muscle–blood biomarker was computed on each sample, and the resulting distribution of values *z*-normalized within each cohort and values from the two cohorts combined for subsequent analysis.

Two multivariate linear regression analyses were performed: first, modelling the *z*-normalized FSHD muscle–blood biomarker across all 83 samples as a function of FSHD status, sex, age and cohort and, second, modelling the *z*-normalized FSHD muscle–blood biomarker across the 54 FSHD samples as a function of Lamperti score, sex, age and cohort. Significance of each coefficient was assessed at *P* < 0.05.

The 54 FSHD samples were split into two groups on the basis of age: 26 patients had ages below the cohort mean (49.6 years) and 28 patients above. These subsets were analysed separately, via multivariate regression analyses modelling the FSHD muscle–blood biomarker as a function of mild/severe FSHD (defined as Lamperti scores 1–7 for mild and 8–15 for severe), age and sex, and significance of each coefficient was assessed at *P* < 0.05. Receiver operating characteristic (ROC) curves and Area under the curve (AUC) calculations were performed using the ‘pROC’ package in R^42^ to assess the strength of the FSHD muscle–blood biomarker as a classifier of mild/severe FSHD across all 54 patients and across the 28 older patients separately.

### Gene Set Enrichment Analysis of the FSHD muscle–blood biomarker

Gene Set Enrichment Analysis (GSEA) was performed separately on the 64 PAX7 upregulated and 79 PAX7 downregulated genes comprising the FSHD muscle–blood biomarker, via Fisher’s exact test, to determine overlap with gene sets defined by the Molecular Signatures Database.^43^

### Statistical analysis

All statistical analysis was performed in R using the approaches detailed above.

## Results

### Clinical assessments correlate strongly in FSHD

Forty-nine individuals were investigated: 25 FSHD1 patients, 1 FSHD2 patient and 23 control individuals (Supplementary Fig. 1). Clinical variables obtained included age, sex and MRC sum score for 12 muscle groups. For FSHD patients, severity indicators were also measured including the following: D4Z4 array length (for FSHD1 patients), Ricci clinical severity score,^35^ Lamperti clinical severity score,^36^ disease duration and LLFF assessed by MRI and MVC of TA (Supplementary Table 1). We also provide the single fat fraction of each muscle (section) and the area of each single muscle alongside weighted average fat fraction and LLFF (Supplementary Table 2).

Considering the entire cohort, there were no significant differences in sex (logit regression *P* = 0.31, FSHD 46% male, control 61% male, Table 1); however, age at examination was significantly higher in FSHD patients versus controls (Wilcoxon *P* = 2.9 × 10^−3^, Table 1). Total MRC sum score was higher in controls (all scored 60/60, Wilcoxon *P* = 3.0 × 10^−9^, Table 1).

**Table 1:** Summary of clinical variables associated with FSHD patients and control individuals

	All samples			Patients providing muscle biopsies			Patients providing PBMCs		
	Control	FSHD	*P-*value	Control	FSHD	*P-*value	Control	FSHD	*P-*value
Number of participants	23	26		11	24		14	15	
Mean age at examination (years)	38.3 (21–65)	50.3 (30–63)	2.90E^−03^	31.6 (21–63)	49.8 (30–63)	5.20E^−03^	32.2 (26–51)	49.5 (30–65)	0.054
Sex (% male)	60.8	46.1	0.31	81.8	45.8	0.047	60	37.5	0.38
Mean MRC muscle grade (max 60)	60 (60–60)	51.6 (26–60)	3.04E^−09^	60 (60–60)	51.25 (26–60)	1.10E^−05^	60 (60–60)	51.9 (26–60)	1.50E^−05^
Mean number of D4Z4 units		6.96 (3–9)			7.0 (3–9)			6.6 (3–9)	
Mean Ricci clinical score (max 10)		4.92 (2–8)			4.96 (2–8)			4.4 (2–8)	
Mean Lamperti clinical score (max 15)		5.81 (1–11)			6 (1–11)			5.1 (1–11)	
Mean disease duration (years)		24 (2–52)			24.4 (2–52)			22.5 (5–52)	
Mean LLFF (%)		14.0 (2.9–56.3)			14.4 (2.9–56.3)			15.3 (3.3–31.2)	
Mean MVC of TA (*n*)		69.6 (9.2–201.0)			69.8 (9.2–201.0)			66.8 (9.2–162.0)	

Mean values for each variable are presented alongside the range in brackets. For age at examination and MRC muscle grade, *P*-value is the significance of a Wilcoxon test comparing control and FSHD-affected individuals in the three groups. For percentage male, a *P*-value represents significance of logit regression comparing sex distribution in control and FSHD-affected individuals across the three groups.

The FSHD severity indicators encompassed three clinical assessments (Ricci, Lamperti and MRC sum scores), an objective assessment of muscle strength (MVC of TA) and the three variables shown to correlate with clinical severity (LLFF, disease duration and D4Z4 array length). Cross-validation of these indicators had not previously been performed in FSHD.

**Figure 1:**
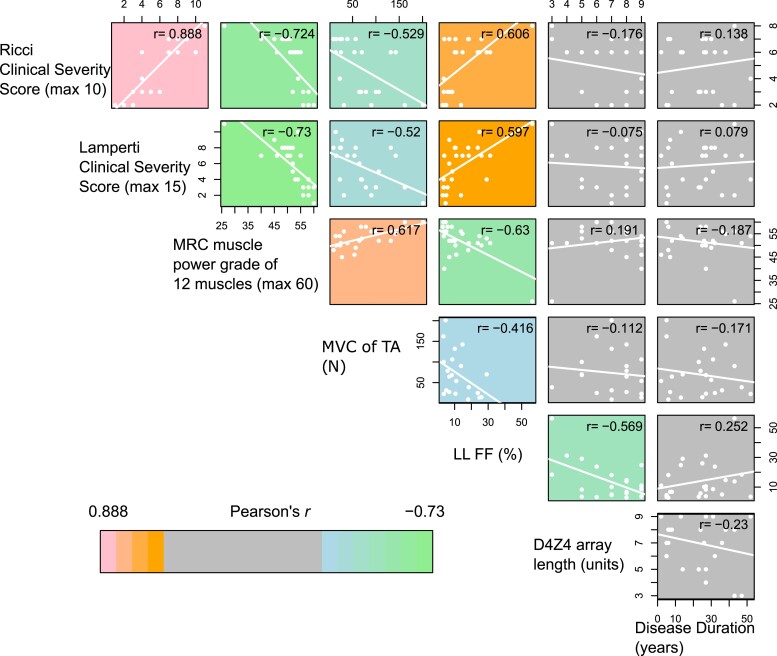
**Correlation between measures of FSHD clinical severity.** Scatter plots display comparisons of FSHD clinical severity scores: Ricci, Lamperti and MRC sum scores, MVC of TA, LLFF, D4Z4 array length (in FSHD1 individuals) and disease duration. Comparisons with non-significant associations are coloured grey, while positive associations are coloured pink to orange and negative associations green to blue in order of significance (assessed at the 5% level). Pearson’s *r* for each comparison is displayed; *n* = 25 (FSHD1); *n* = 1 (FSHD2).

All three clinical assessments correlated strongly with one another (Ricci versus Lamperti score: Pearson’s *r* = 0.89, *P* = 1.4 × 10^−9^; Ricci versus MRC sum score: Pearson’s *r* = −0.72, *P* = 2.9 × 10^−5^; Lamperti versus MRC sum score: Pearson’s *r* = −0.73, *P* = 2.3 × 10^−5^, Fig. 1). The three clinical severity scores also correlated with the objective measure of muscle strength i.e. MVC of TA (Ricci score versus MVC of TA: Pearson’s *r* = −0.53, *P* = 0.011; Lamperti score versus MVC of TA: Pearson’s *r* = −0.52, *P* = 0.013; MRC sum score versus MVC of TA: Pearson’s *r* = 0.62, *P* = 0.0022, Fig. 1), as well as with LLFF (Ricci score versus LLFF: Pearson’s *r* = 0.61, *P* = 0.0013; Lamperti score versus LLFF: Pearson’s *r* = 0.60, *P* = 0.0016; MRC sum score versus LLFF: Pearson’s *r* = −0.63, *P* = 7.4 × 10^−4^, Fig. 1). D4Z4 array length was inversely associated with LLFF (Pearson’s *r* = −0.57, *P* = 0.0037, Fig. 1), consistent with known association between shorter D4Z4 array lengths and more severe pathology,^48^ although not with Ricci, Lamperti, MRC sum score nor MVC of TA. Self-reported FSHD disease duration did not correlate with any other markers of clinical severity (Fig. 1).

Multivariate linear regression showed Ricci and Lamperti scores were both higher in older male patients (Ricci score: age at examination *P* = 0.012, sex *P* = 0.033; Lamperti score: age at examination *P* = 0.047, sex *P* = 0.038). The MRC sum score was also significantly lower in male patients (*P* = 0.042) but was not associated with age at examination. No other clinical variables demonstrated association between age and sex.

### PAX7 target gene repression discriminates control, non-inflamed (TIRM^−^) and inflamed (TIRM^+^) FSHD muscles

Muscle biopsies were obtained from 35/49 individuals for RNA-sequencing. Twenty-three FSHD1 patients and 1 FSHD2 patient underwent MRI-guided muscle biopsies with samples taken from a TIRM^−^ (23/24 vastus lateralis) and a TIRM^+^ muscle (15/24 gastrocnemius medialis) from each patient. Eleven control individuals also underwent muscle biopsy of vastus lateralis. FSHD patients who underwent muscle biopsy were older than controls and showed a male sex bias (Table 1; Supplementary Table 1).

Five known FSHD biomarkers^17-19,25^ were considered in our muscle biopsy samples. Three biomarkers are based on DUX4 target gene expression: our Choi *et al*., a set of 212 ‘early’ DUX4 target genes^18,37^; Geng *et al*., a set of 165 ‘late’ DUX4 target genes^18,38^; and the Yao *et al*., a set of 114 ‘late’ DUX4 target genes.^39,49^ The fourth FSHD biomarker is based on PAX7 target gene repression that we derived from 311 upregulated and 290 downregulated PAX7 target genes.^18^ The final biomarker was our Lymphoblast score, derived from 237 genes found to be upregulated in FSHD lymphoblastoid cell lines compared to controls.^19^

**Figure 2:**
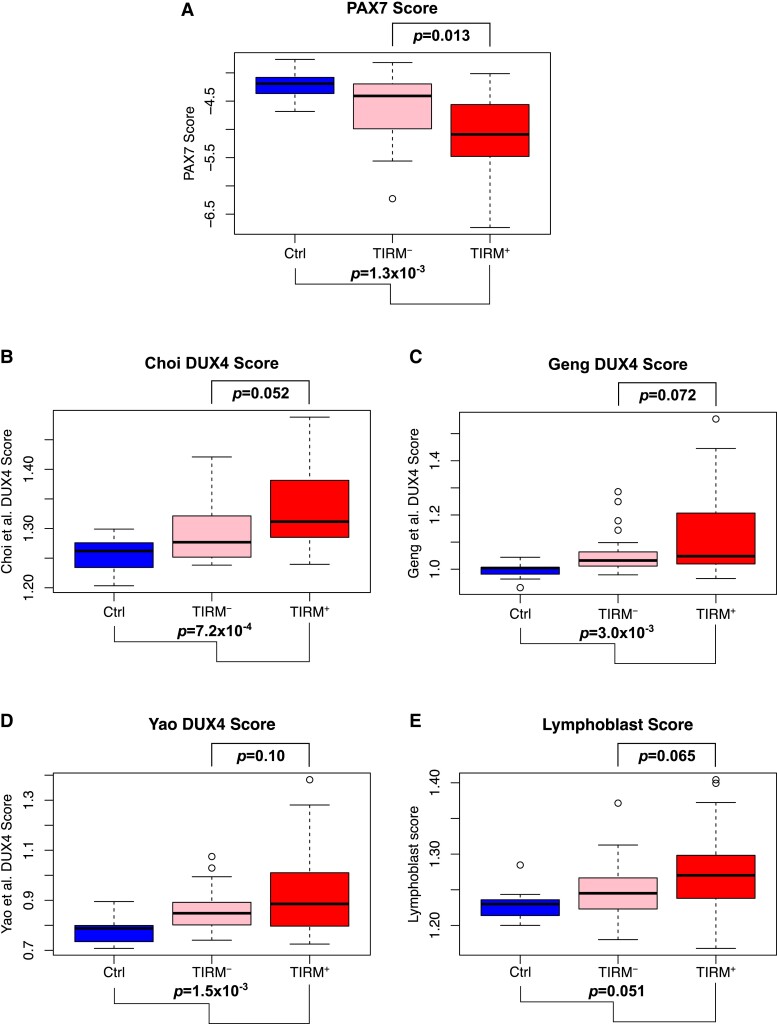
**Analysis of FSHD biomarkers in inflamed (TIRM^+^), non-inflamed (TIRM^−^) and control muscle and PBMCs.** (**A–E**) Boxplots demonstrate (**A**) the PAX7 target gene score, (**B**) Choi *et al*. early DUX4 target genes, (**C**) Geng *et al*. and (**D**) Yao *et al*. late DUX4 target genes and (**E**) the Lymphoblast score in muscle biopsies from 11 control individuals and 24 FSHD TIRM^−^ and TIRM^+^ samples. Box represents the interquartile range (IQR), with median indicated by a line. Whiskers denote min [1.5∗IQR, max (observed value)]. Multivariate regression *P*-values adjusting for age and sex are shown for each biomarker as a correlate of control versus TIRM^−^/TIRM^+^ status below each plot and separately also adjusting for isogenic TIRM^−^ and TIRM^+^ samples above each plot.

As expected, PAX7 target gene repression proved a clear biomarker of FSHD status, discriminating FSHD and control muscle biopsies (multivariate regression: PAX7 score versus control/TIRM^−^/TIRM^+^ status adjusting for age and sex, *P* = 0.0013). Our PAX7 biomarker also discriminated isogenic TIRM^−^ and TIRM^+^ FSHD samples (multivariate regression on FSHD samples: PAX7 score versus TIRM status adjusting for age, sex and patient, *P* = 0.013) (Fig. 2A). The three DUX4 target gene biomarkers all discriminated FSHD from controls; however, none discriminated between isogenic TIRM^−^ and TIRM^+^ FSHD samples on paired analysis at the 5% significance level (Fig. 2B–D).

Our Lymphoblast score correlates with the degree of inflammation in FSHD muscle biopsies.^19^ Here, the Lymphoblast score was not a significant biomarker of FSHD muscle compared to control, nor was it able to discriminate between isogenic TIRM^−^ and TIRM^+^ FSHD muscle samples on paired analysis (Fig. 2E).

Associations between the five FSHD scores and disease severity are summarized in Table 2.

**Table 2:** Summary of comparisons performed: PAX7 score and Lymphoblast score show association between FSHD muscle and PBMC samples and correlate with severity assessments

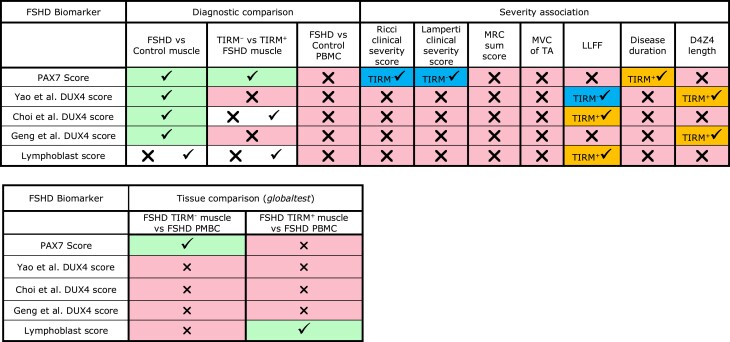

Tick on green denotes significant association at the 5% level, while a cross on pink denotes no association. Both a cross and a tick on white refer to non-significance of multivariate regression but significance of ‘globaltest’ analysis. Biomarkers achieving severity association in only TIRM^−^ (tick, blue) or TIRM^+^ (tick, yellow) muscle biopsies are indicated.

A recent analysis investigated the PAX7 and DUX4 target gene biomarker sets in FSHD PBMCs via the ‘globaltest’ package.^31^ Unlike our approach, ‘globaltest’ does not give a single sample score for use as a biomarker but determines the association between a set of genes and given phenotype. We assessed the gene sets on which the five biomarkers are based using ‘globaltest’ methodology, which did not change our findings for the PAX7, Geng *et al*. and Yao *et al*. DUX4 target genes. However, the Choi *et al*. early DUX4 target genes were also significantly associated with TIRM status among isogenic FSHD muscle samples (adjusting for age and sex), and the genes comprising the Lymphoblast score were significantly associated with FSHD versus control status as well as TIRM status in isogenic muscle samples (Table 2; Supplementary Table 3). This indicates that our biomarker score construction is suboptimal for our Choi *et al*. DUX4 target gene signature and our Lymphoblast signature.

TIRM^+^ and TIRM^−^ muscle biopsies were not all sampled from the same muscle. To determine the impact of anatomical muscle on the FSHD biomarkers, we performed a regression analysis separately on TIRM^−^ and TIRM^+^ FSHD biopsies, modelling each biomarker as a function of anatomical muscle, age and sex. We found no association between PAX7 target gene repression or the Lymphoblast score and muscle across TIRM^+^ and TIRM^−^ samples and no association between the Choi *et al*. DUX4 target score and anatomical muscle across TIRM^−^ samples. However, the Geng *et al*. and Yao *et al*. DUX4 target scores both showed elevated levels in the single *vastus intermedius* TIRM^−^ sample compared to the 23 *vastus lateralis* TIRM^−^ samples. The Yao *et al*. DUX4 target score also demonstrated elevated levels on the single *soleus* and *sartorius* TIRM^+^ samples compared to the other TIRM^+^ samples, while the Geng *et al*. DUX4 score was elevated on the *sartorius* TIRM^+^ sample and the Choi *et al*. DUX4 score on the *soleus* TIRM^+^ sample, compared to other TIRM^+^ muscles. To limit impact of anatomical muscle on biomarker discrimination, data were re-analysed. The TIRM^−^*vastus intermedius* sample was excluded from comparison involving the Yao *et al*. and Geng *et al*. scores, the *sartorius* TIRM^+^ sample was excluded from comparisons involving the Yao *et al*. and Geng *et al*. scores and the *soleus* sample was excluded from comparisons involving the Yao *et al*. and Choi *et al*. scores. Despite this, the significance of all results remained unchanged.

### PAX7 target gene repression correlates with Ricci and Lamperti clinical severity scores

**Figure 3:**
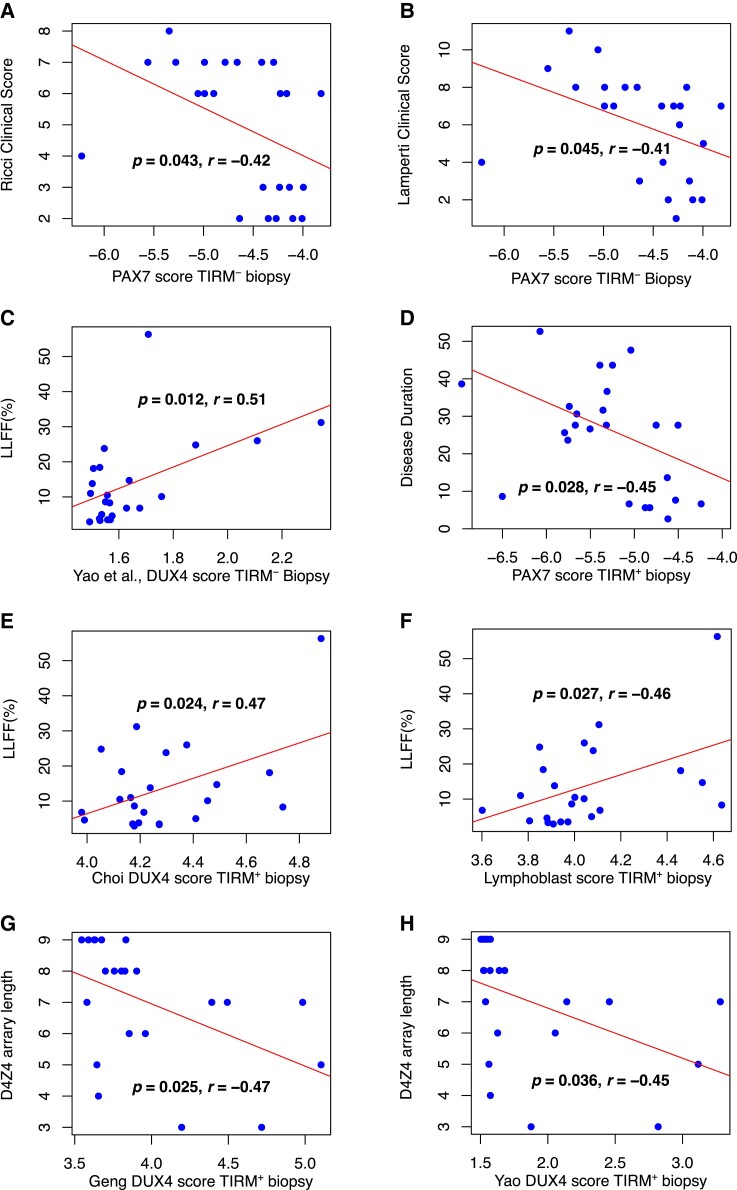
**Association between FSHD biomarkers and clinical severity variables in muscle biopsies.** (**A**, **B**) Scatter plots show (**A**) Ricci and (**B**) Lamperti clinical severity scores against PAX7 target gene expression in TIRM^−^ FSHD muscle biopsies from 24 patients. (**C**) LLFF is plotted against Yao *et al*. late DUX4 target biomarker in the same 24 TIRM^−^ FSHD muscle biopsies. (**D**) Disease duration against PAX7 target gene expression in TIRM^+^ FSHD muscle biopsies from 24 patients. (**E**, **F**) LLFF is plotted against (**E**) Choi *et al*. early DUX4 target biomarker and (**F**) the Lymphoblast score, in 24 TIRM^+^ FSHD muscle biopsies. (**G**, **H**) D4Z4 array length is plotted against (**G**) Geng *et al*. and (**H**) Yao *et al*. late DUX4 target gene biomarkers in 23 FSHD1 TIRM^+^ muscle biopsies. Pearson’s *r* and associated *P*-value are given alongside a line of best fit.

We next considered associations between FSHD biomarkers and clinical severity. Across TIRM^−^ FSHD muscle, PAX7 target gene repression correlated with Ricci and Lamperti clinical severity scores (Ricci: Pearson’s *r* = −0.42, *P* = 0.043; Lamperti: Pearson’s *r* = −0.41, *P* = 0.045, Fig. 3A and B). The Yao *et al*. late DUX4 target genes correlated with LLFF in TIRM^−^ FSHD samples (Pearson’s *r* = 0.51, *P* = 0.012, Fig. 3C).

Across TIRM^+^ FSHD muscle, PAX7 target gene repression correlated with disease duration (Pearson’s *r* = −0.45, *P* = 0.028, Fig. 3D), consistent with our demonstration that PAX7 target genes are progressively repressed in biopsies from the same patients taken 1 year apart.^25^ Both Choi *et al*. early DUX4 target genes and the Lymphoblast score correlated with LLFF (Choi *et al*.: Pearson’s *r* = 0.47, *P* = 0.024, Fig. 3E; Lymphoblast score: Pearson’s *r* = 0.46, *P* = 0.027, Fig. 3F). Lastly, late DUX4 target genes of Geng *et al*. and Yao *et al*. inversely correlated with D4Z4 array length (Geng *et al*.: Pearson’s *r* = −0.47, *P* = 0.025, Fig. 3G; Yao *et al*.: Pearson’s *r* = −0.45, *P* = 0.036, Fig. 3H).

### Generating an effective FSHD blood biomarker

PBMCs were isolated from 14 FSHD1 and 1 FSHD2 patients (12 FSHD1 and the FSHD2 also underwent muscle biopsies) and 14 control individuals (two also underwent muscle biopsy). None of the five biomarkers discriminated FSHD and control PBMCs after adjustment for age and sex either via multivariate regression or ‘globaltest’ (Table 2; Supplementary Table 3).

**Figure 4:**
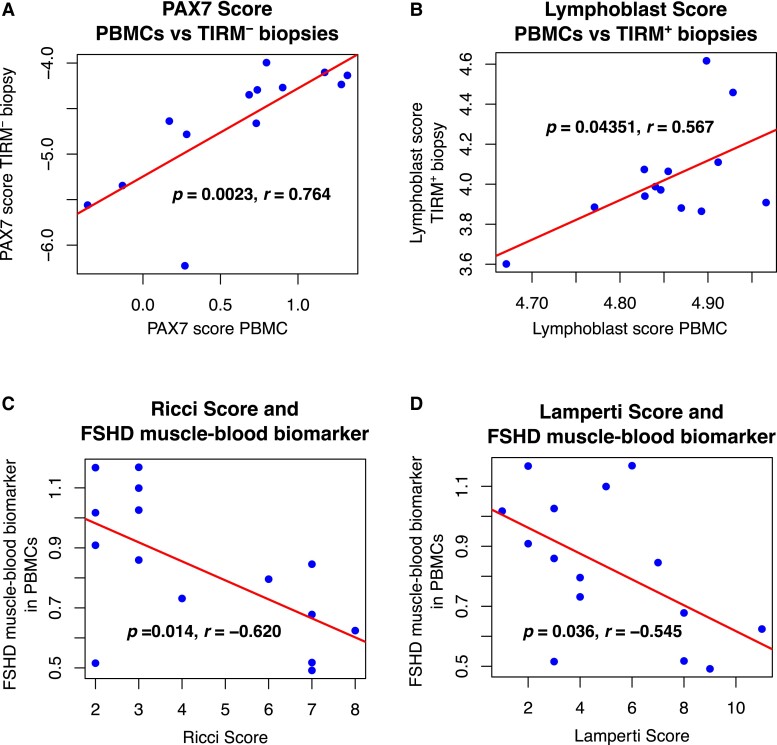
**Association between PAX7 and Lymphoblast score across muscle and blood samples and association between FSHD muscle–blood biomarker in PBMCs and clinical severity measures.** (**A**, **B**) Scatter plots show (**A**) PAX7 target gene biomarker levels in 13 TIRM^−^ FSHD muscle biopsies against levels in 13 isogenic PBMCs and (**B**) the Lymphoblast score levels in 13 TIRM^+^ FSHD muscle biopsies against levels in 13 isogenic PBMCs. (**C**, **D**) The 143-gene FSHD muscle–blood biomarker in PBMCs is plotted against (**C**) Ricci and (**D**) Lamperti clinical severity scores in 15 FSHD PBMCs. Pearson’s *r* and associated *P*-value are provided, alongside line of best fit.

We considered each of our five biomarkers in the 13 FSHD PBMC samples with isogenic muscle biopsies (Supplementary Fig. 1) and investigated whether the level of the biomarkers correlated between muscle and blood. We found two significant positive correlations: between PAX7 target gene repression in PBMCs and TIRM^−^ FSHD muscle biopsies (*P* = 0.002, Pearson’s *r* = 0.76, Fig. 4A) and between the Lymphoblast score in PBMCs and TIRM^+^ FSHD muscle biopsies (*P* = 0.04, Pearson’s *r* = 0.57, Fig. 4B). None of the DUX4 target gene expression biomarkers correlated in levels between muscle and PBMCs.

The levels of PAX7 target genes and Lymphoblast score in PBMCs, however, did not associate with any clinical variables. Both these biomarkers associate with FSHD status in muscle biopsies and correlate with clinical variables in FSHD muscle biopsies. The lack of association of the full biomarkers with clinical severity in FSHD PBMCs may be attributable to a lack of clinical relevance of only a subset of the biomarker genes in peripheral blood. If this is the case, a core of severity-associated genes in PBMCs may be extracted from refinement of these biomarkers.

We thus performed a refinement of these two signatures to reduce noise in the association between the biomarker level in muscle and clinical variables.

For each of the 237 genes of the Lymphoblast score in TRIM^−^ and TRIM^+^ muscle, we identified which had expression levels significantly correlated with the associated clinical variable of LLFF and identified 44/237 genes. A Refined Lymphoblast score biomarker was set as the mean expression of the 44 genes in a given sample. This Refined Lymphoblast score had no association with any clinical variables in PBMCs (Table 3).

**Table 3:** Biomarker refinement: the 143-gene FSHD muscle–blood biomarker correlates with severity in FSHD muscle and PBMCs

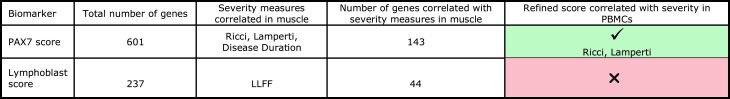

A tick in a green box denotes significant association at the 5% level between the refined score and the listed severity measure, while a cross in a pink box denotes no association.

We next considered the 601 genes comprising the PAX7 score (311 upregulated and 290 downregulated PAX7 target genes).^18^ For each of the PAX7 target genes in TIRM^−^ and TIRM^+^ muscle, we identified which had expression levels significantly correlated with the associated clinical variables of the Ricci or Lamperti severity scores or disease duration. This analysis identified 143 genes (64/311 upregulated and 79/290 downregulated, Supplementary Table 4).

### The FSHD muscle–blood biomarker correlates with clinical severity in FSHD muscle and PBMCs

A refined PAX7 target gene biomarker was defined as the *t*-statistic evaluating the difference between expression of the 64 upregulated and the 79 downregulated PAX7 target genes associated with clinical severity in muscle in a given sample. This refined PAX7 target gene biomarker showed significant association between its level in PBMCs and the Ricci or Lamperti clinical severity scores, independent of age or sex (Ricci score: *P* = 0.014, *r* = −0.62; Lamperti score: *P* = 0.036, *r* = −0.55, Fig. 4C and D and Table 3). PAX7 does not have a defined role in blood cells, and the 143 genes identified are not necessarily direct target genes of PAX7 and may be regulated in multiple ways. We therefore named our refined PAX7 biomarker the ‘FSHD muscle–blood biomarker’.

**Figure 5:**
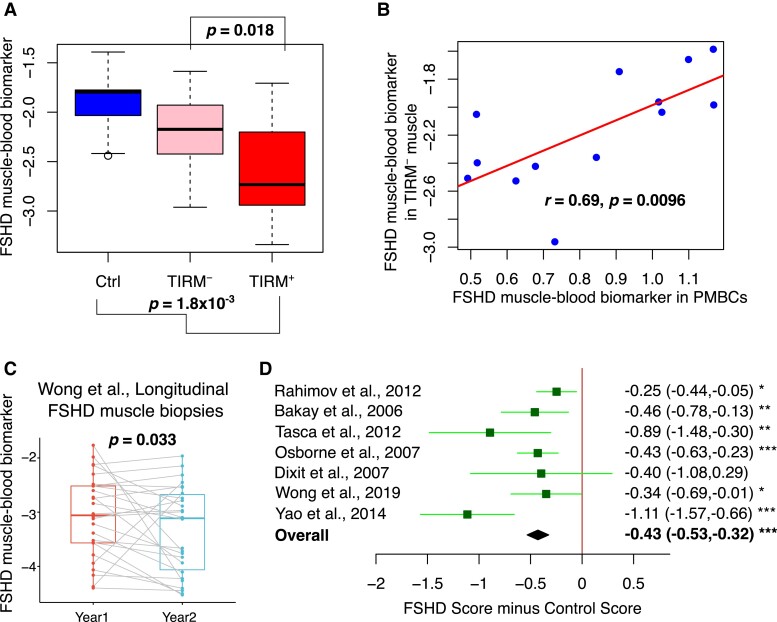
**Validation of the 143-gene FSHD muscle–blood biomarker.** (**A**) Boxplot displays the 143-gene FSHD muscle–blood biomarker values in muscle biopsies from 11 control individuals and 24 FSHD TIRM^−^ and TIRM^+^ samples. Box represents the interquartile range (IQR), with median indicated by a line. Whiskers denote min [1.5∗IQR, max (observed value)]. Multivariate regression *P*-values adjusting for age and sex are shown for the biomarker as a correlate of a variable describing control, TIRM^−^ and TIRM^+^ status computed across all samples below the plot and separately considering only FSHD samples and adjusting for isogenic TIRM^−^ and TIRM^+^ samples above the plot. (**B**) Scatter plot displays the FSHD muscle–blood biomarker in 13 TIRM^−^ FSHD muscle biopsies against levels in 13 isogenic PBMCs. Pearson’s *r* and associated *P*-value are given alongside a line of best fit. (**C**) Boxplot demonstrates the refined 143-gene PAX7 score in 26 isogenic Year 1 and Year 2 FSHD muscle biopsy samples described by Wong *et al*.^22^ Lines demonstrate connections between paired samples, and a paired Wilcoxon test *P*-value is displayed. Box represents the IQR, with median indicated by a line. Whiskers denote min [1.5∗IQR, max (observed value)]. (**D**) Forest plot displays the significance of the FSHD muscle–blood biomarker as a discriminator of FSHD muscle biopsies in seven independent microarray or RNA-seq data sets [*n* = 130 (FSHD); *n* = 98 (control)]. Green boxes denote mean difference in the FSHD muscle–blood biomarker between FSHD and control muscle biopsies, and whiskers denote 95% confidence interval. A vertical line denotes a score difference of 0, and data sets where the whiskers cross this line have not attained significance at *P* < 0.05 (as assessed by Wilcoxon test). Numerical values for mean score difference and confidence interval are displayed for each data set to the right of the plot with significance denoted by asterisks where * denotes *P* < 0.05, ** denotes *P* < 0.01 and *** denotes *P* < 0.001. Overall estimate is displayed as a black diamond and was computed using a random effects model with significance assessed via Fisher’s combined test.

As per the full PAX7 score, our 143-gene FSHD muscle–blood biomarker can discriminate control, TIRM^−^ and TIRM^+^ samples (Fig. 5A) and correlates in levels between PBMCs and TIRM^−^ muscle biopsies (Fig. 5B). Importantly, using the longitudinal, transcriptomic muscle biopsy data set published by Wong *et al*.,^22^ the FSHD muscle–blood biomarker discriminates Year 1 from Year 2 FSHD muscle biopsy samples (paired Wilcoxon *P* = 0.03, Fig. 5C), as does the full PAX7 score.^25^ Finally, the FSHD muscle–blood biomarker discriminates FSHD from control samples on meta-analysis and on 6/7 independent FSHD muscle biopsy transcriptomic data sets (Fisher’s combined *P* = 3.4 × 10^−10^, Fig. 5D), a performance comparable to the full PAX7 score (significant on 7/7 data sets) (summarized in Table 4).

**Table 4:** Summary of the diagnostic, progression and severity associations of PAX7 target gene repression score and FSHD muscle–blood biomarker

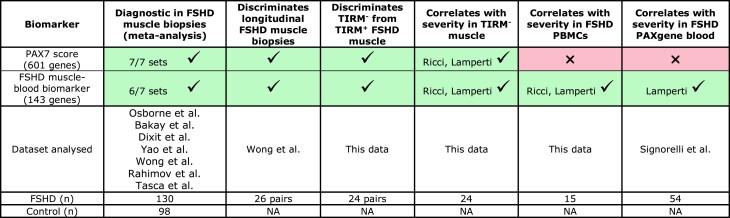

Tick on green denotes significant association at the 5% level and the listed severity measure, while a cross on pink denotes no association, together with data sets analysed.

GSEA via Fisher’s exact test on genes comprising the FSHD muscle–blood biomarker was performed. Genes with expression suppressed in muscle and blood of patients with severe disease showed enrichment for pathways previously implicated in FSHD: including targets of the DREAM complex,^50^ neuronal gene sets,^51^ Wnt signalling,^52^ hypoxia response^18^ and hormone response and regulation,^53,54^ including 17β-hydroxysteroid dehydrogenase 8 targets, and oestradiol response^55^ (Supplementary Table 5). Genes upregulated in muscle and blood of FSHD patients with severe disease showed enrichment for epithelial to mesenchymal transition,^56^ Wnt signalling,^52^ TGF-β signalling^57^ and vasculature development^47^ (Supplementary Table 5).

### The FSHD muscle–blood biomarker validates as a blood biomarker of clinical severity in an independent data set

To validate our FSHD muscle–blood biomarker as a circulating measure of clinical severity in FSHD, we considered the data set of Signorelli *et al*.^31^ describing RNA-sequencing of PAXgene blood from 54 FSHD patients and 29 controls. The data set comprises two independent cohorts processed separately. The Nijmegen cohort comprises 39 FSHD and 11 control samples. The Newcastle cohort comprises 15 FSHD and 18 control samples. Clinical annotations for each sample included patient age, sex and the Lamperti clinical severity score. Raw read counts were obtained from the authors, data were normalized separately in each cohort, and values for the FSHD muscle–blood biomarker were computed for each sample and *z*-normalized within cohort.

**Figure 6:**
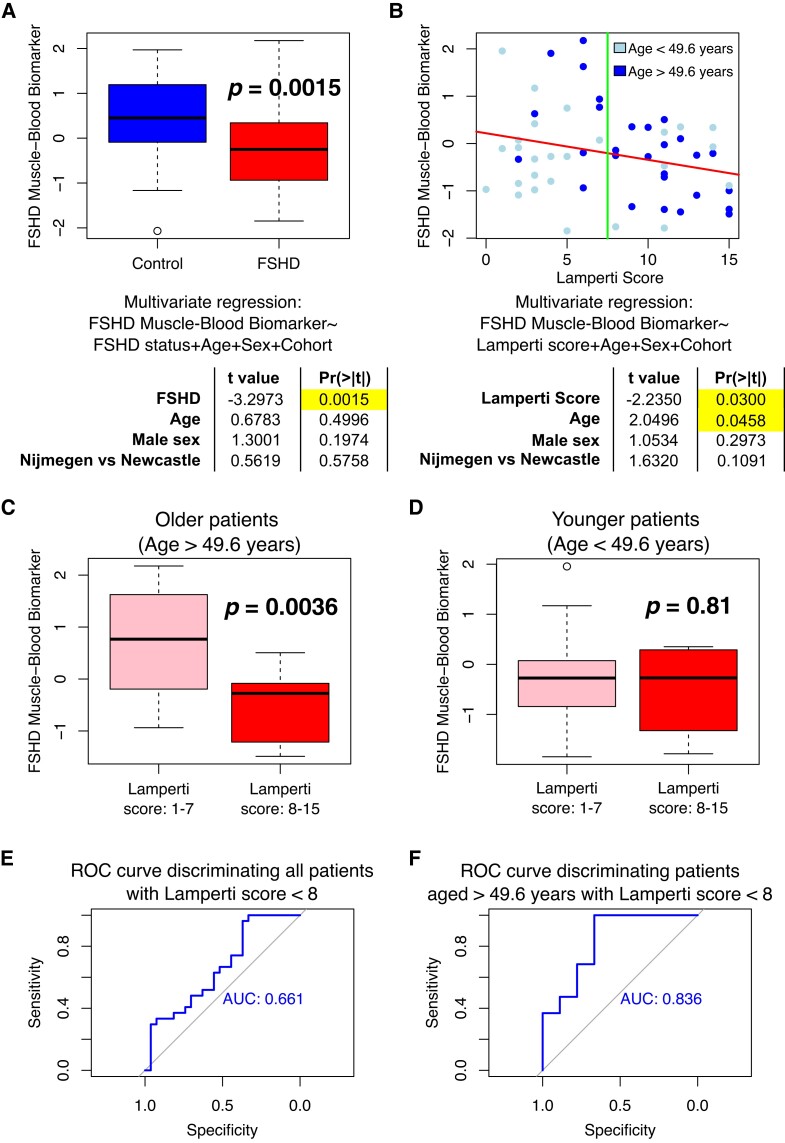
**Investigation of the FSHD muscle–blood biomarker in an independent cohort of 54 FSHD and 29 control peripheral blood samples.** (**A**) Boxplot demonstrates the 143-gene FSHD muscle–blood biomarker, *z*-normalized within cohort in peripheral blood RNA-seq samples from 54 FSHD patients and 29 controls described by Signorelli *et al*.^31^ Box represents the interquartile range (IQR), with the median indicated by a line. Whiskers denote min [1.5∗IQR, max (observed value)]. Results are presented of multivariate linear regression, modelling the FSHD muscle–blood biomarker across 54 FSHD samples and 29 controls as a function of FSHD status, age, sex and cohort. (**B**) Scatter plot displays the FSHD muscle–blood biomarker, *z*-normalized within cohort in peripheral blood RNA-seq samples of 54 FSHD patients against Lamperti score. Light blue points denote patients below and dark blue for patients above the mean cohort age of 49.6 years. Red line of best fit is presented, and a green line corresponds to Lamperti score = 7.5, separating patients with mild and severe FSHD. Results of multivariate linear regression, modelling the FSHD muscle–blood biomarker across 54 FSHD samples as a function of Lamperti score, age, sex and cohort are given. (**C**, **D**) Boxplots display the FSHD muscle–blood biomarker in (**C**) 9 FSHD patients with Lamperti scores 1–7 and 19 FSHD patients with Lamperti scores 8–15 aged >49.6 years and (**D**) 18 FSHD patients with Lamperti scores 1–7 and 8 FSHD patients with Lamperti scores 8–15 aged <49.6 years. Box represents the IQR, with median indicated by a line. Whiskers denote min [1.5∗IQR, max (observed value)]. Multivariate regression analysis of FSHD muscle–blood biomarker as a function of FSHD severity (Lamperti scores 1–7 versus 8–15), age, sex and cohort was performed separately in younger and older groups, and *P*-values for the severity coefficient are displayed on each plot. (**E**, **F**) Receiver operating characteristic (ROC) curves and associated Area under the curve (AUC) values for the FSHD muscle–blood biomarker as a marker of FSHD severity (Lamperti scores 1–7 versus 8–15) are displayed for (**E**) all 54 FSHD samples and (**F**) 28 FSHD patients aged >49.6 years.

The FSHD blood–muscle biomarker was not a significant indicator of FSHD status on our data set of 15 FSHD and 14 control PBMCs, after adjusting for age and sex (multivariate regression *P* = 0.2) but demonstrated lower average values on FSHD samples (FSHD mean: 0.83, range: 0.49, 1.16; control mean: 0.93, range: 0.67, 1.2). To determine the association of the FSHD muscle–blood biomarker with FSHD status in the larger data set of Signorelli *et al*.,^31^ multivariate regression analysis was performed modelling the *z*-normalized FSHD muscle–blood biomarker values as a function of FSHD status, age, sex and cohort. The FSHD muscle–blood biomarker was significantly lower on FSHD blood samples versus controls, independently of age, sex and cohort (*P* = 0.002, Fig. 6A).

The FSHD muscle–blood biomarker significantly correlated with the Lamperti score in our data set of 15 FSHD PBMCs. To determine the association of the FSHD muscle–blood biomarker and Lamperti score in the larger blood sample data set of Signorelli *et al*.,^31^ multivariate regression analysis was performed modelling the *z*-normalized FSHD muscle–blood biomarker values as a function of Lamperti score, age, sex and cohort. The FSHD muscle–blood biomarker significantly negatively correlated with the Lamperti score independently of age, sex and cohort (*P* = 0.030, Fig. 6B), confirming our biomarker as a measure of clinical severity in blood samples (Table 4).

To ascertain whether this association is attributable to chance, we performed resampling, selecting 1000 independent, random, non-overlapping sets of 143 genes, divided into 64 upregulated and 79 downregulated targets. For each random gene set, we performed multivariate analysis as above to determine association with FSHD status and severity in the Signorelli *et al*.^31^ data set. Only 32/1000 random signatures could discriminate FSHD from control samples as well as the true FSHD muscle–blood biomarker (*P* = 0.032), and only 6/1000 showed as strong an association with the Lamperti score (*P* = 0.006).

Curiously, the FSHD muscle–blood biomarker positively correlated with patient age, independently of Lamperti score, sex and cohort (*P* = 0.046, Fig. 6B). This is unexpected as Lamperti score and patient age are positively correlated in the Signorelli *et al*.^31^ data set (Pearson’s *r* = 0.35, *P* = 0.009). It would be anticipated that the FSHD muscle–blood biomarker would have unidirectional association with both variables. This indicates that the FSHD muscle–blood biomarker has different associations with the Lamperti score in older versus younger patients. To investigate, we split the FSHD patients in the Signorelli *et al*.^31^ data set into two groups based on mean age of FSHD patients in the cohort (49.6 years): an older group (28 patients aged >49.6 years) and a younger group (26 patients aged <49.6 years). The younger and older groups showed no significant difference in sex distribution (*P* = 0.74) or Nijmegen/Newcastle cohort (*P* = 0.64). As expected, the older group displayed significantly higher Lamperti scores (*P* = 0.002, younger group mean: 5.8, range: 0–15; older group mean: 9.4, range: 2–15, Fig. 6B). Association between the FSHD muscle–blood biomarker and the Lamperti score is driven by the older patient cohort, with the score robustly able to discriminate older patients with mild FSHD (Lamperti scores: 1–7) from those with more severe FSHD (Lamperti scores: 8–15) (*P* = 0.004, Fig. 6C) but showing no discrimination of mild versus severe in younger patients (*P* = 0.81, Fig. 6D). As a binary classifier of mild versus severe FSHD, the FSHD muscle–blood biomarker obtains modest accuracy on the full Signorelli *et al*.^31^ data set (AUC = 0.661, Fig. 6E) but robust classification on the older FSHD patients (AUC = 0.836, Fig. 6F).

## Discussion

We investigated transcriptomic biomarkers in RNA-sequencing data from isogenic non-inflamed (TIRM^−^) and inflamed (TIRM^+^) muscle biopsies and PBMCs from clinically characterized FSHD patients, alongside matched controls. Our PAX7 target gene repression biomarker^18^ discriminates control, non-inflamed and inflamed FSHD muscle, while the discriminatory power of DUX4 target gene signatures is limited to distinguishing control from FSHD muscle (Table 2). We also developed a new 143-gene FSHD muscle–blood biomarker that shows levels correlated between isogenic TIRM^−^ muscle and PBMCs. Importantly, the level of our FSHD muscle–blood biomarker in both TIRM^−^ muscle and PBMCs correlates with Ricci and Lamperti measures of clinical severity (Table 3). By analysing an independent data set describing RNA-sequencing of peripheral blood for 54 clinically characterized FSHD patients and 29 matched controls,^31^ we validated our FSHD muscle–blood biomarker as a circulating measure of clinical severity (Table 4), with particular strength in older patients.

Our FSHD muscle–blood biomarker is the first circulating indicator of FSHD clinical severity with direct correlation to transcriptomic changes in patient muscle, valid on a cohort level. The signature could be developed into a minimally invasive tool for routine use in clinic and monitoring patients in trials. The 143 genes comprising the FSHD muscle–blood biomarker represent an important discovery set for FSHD pathomechanisms and therapeutic targets, valid in both muscle and blood.

FSHD displays both inter-patient and intra-patient heterogeneity, with the degree of muscle weakness and affected muscle distribution varying dramatically. This inhibits development of reliable biomarkers based on single muscle biopsies from patients. FSHD also has systemic elements such as inflammation, suggesting consideration of immune cells, both peripheral and in inflamed tissue, may yield clinically relevant information. Our well-controlled data set takes muscle biopsies from inflamed and non-inflamed regions of two different muscles from each clinically characterized FSHD patient, alongside isogenic peripheral blood, allowing adjustment for heterogeneity and identification of a transcriptomic biomarker valid in multiple tissue types.

Importantly, our FSHD muscle–blood biomarker correlates with robust clinical assessments. Comparative analysis of clinical severity measures for FSHD demonstrated strong concordance between the Ricci^35^ and Lamperti^36^ clinical severity scores, which in turn correlate with objective measures of function in FSHD patients including MVC of TA and LLFF on MRI, confirming the clinical assessments as robust characterizations of FSHD severity.

The data set of Signorelli *et al*.^31^ was used as a validation set for our biomarkers, and we confirmed findings that the full PAX7 target gene score and DUX4 target gene sets are not FSHD blood biomarkers. Signorelli *et al*.^31^ reported no genes with expression levels associated with FSHD status or clinical severity in their discovery analysis when investigating the Nijmegen and Newcastle cohorts separately. In our validation approach to this data, we compute our novel FSHD muscle–blood biomarker separately in each cohort and integrate the independent data sets, facilitating a larger sample size to validate our biomarker as a negative correlate of clinical severity.

We found that the FSHD muscle–blood biomarker has a stronger association with clinical severity in older patients. This may be attributed to several factors, but the FSHD muscle–blood biomarker could have predictive value on FSHD disease progression. If true, in older patients who have reached their potential disease severity, clinical scores and biomarker values may be expected to match closely, while in younger patients who are early in the disease process, low biomarker values may imply a more severe disease progression. There are no patients (young or old) with high biomarker values (normalized biomarker level > 0.567) and severe FSHD (Lamperti scores > 7), implying no patients have been ‘predicted’ a mild score and achieved a severe score. Moreover, patients with low biomarker values and mild FSHD (Lamperti scores < 7) are younger (83% below mean cohort age), suggesting that they may yet attain a ‘predicted’ severe phenotype with age. Conversely, those with low biomarker values and severe FSHD tend to be older (70% above mean cohort age), suggesting that a ‘predicted’ severe phenotype has been achieved by greater age. Validation of the FSHD muscle–blood biomarker as a predictor of severity will require longitudinal studies.

Recently, van den Heuvel *et al*.^20^ performed RNA-sequencing of FSHD and control muscle biopsies from different muscle groups. The authors identified non-overlapping roles for PAX7 and DUX4 target genes, with both biomarkers independently associated with fatty replacement of muscle but only DUX4 target gene expression associated with inflammation,^20^ consistent with our original findings.^17,19^ This emphasizes the importance of studying biomarkers in non-inflamed and inflamed muscle separately. As our study profiles isogenic TIRM^+^/TIRM^−^ muscle biopsies (i.e. from the same FSHD patients), we account for inter-patient heterogeneity in FSHD, allowing deeper analysis of biomarker associations. PAX7 target genes associate with FSHD muscle regardless of inflammatory state and can discriminate TIRM^−^ FSHD muscle from isogenic TIRM^+^. In contrast, DUX4 target genes have discriminatory power limited to separating FSHD muscle from control. van den Heuvel *et al*.^20^ highlighted the importance of minimally invasive biomarkers for FSHD severity, focusing on use of MRI.^20^ We propose a complementary approach, based on gene expression in minimally invasive blood samples.

We found no correlation between DUX4 target gene expression in FSHD muscle and isogenic PBMCs. Our Lymphoblast score comprises genes upregulated in FSHD lymphoblastoid cell lines compared to matched controls^19^ and here demonstrated significant correlation between TIRM^+^ FSHD muscle and PBMCs but not TIRM^−^ biopsies. This is consistent with our previous demonstration that the Lymphoblast score associates with the degree of histological inflammation in FSHD muscle biopsies.^19^ However, the Lymphoblast score could not be further refined into a biomarker of FSHD clinical severity valid in PBMCs, though this may be related to construction of the Lymphoblast score, which was shown suboptimal by ‘globaltest’. Curiously, our PAX7 target gene repression score showed strong correlation in levels between TIRM^−^ FSHD muscle and PBMCs, but not TIRM^+^, supporting a role for PAX7 target genes specifically in non-inflamed muscle. Studies have proposed a role for PAX7 in development of the thymus,^58,59^ and *DUX4* expression has been shown in healthy human thymus,^60^ raising the possibility that the two proteins may interact in lymphoid progenitor tissue and possibly affect epigenetic programming, as has been proposed in muscle.^24^*PAX7* is not known to be expressed in mature immune cells, although *Pax5* is expressed in lymphoid precursors and required for B cell differentiation.^61^

The PAX7 target gene biomarker was refined to the FSHD muscle–blood biomarker, a measure of clinical severity valid in TIRM^−^ muscle and PBMCs. Genes comprising the PAX7 target gene biomarker are not necessarily direct PAX7 targets and likely regulated by multiple transcription factors/other events. GSEA of the 143 genes comprising the FSHD muscle–blood biomarker identified numerous pathways known to be dysregulated in FSHD, including vasculogenesis^47^ and hypoxic response,^62^ indicating that misregulation of these processes in FSHD is not limited to muscle. TGF-β signalling associated with genes upregulated in clinically severe FSHD and is well studied in muscular dystrophy, particularly myostatin, which limits muscle growth via mechanisms directly relevant to FSHD, including direct suppression of PAX7 and activation of p38.^63^ Targeting TGF-β has been considered as a therapy for FSHD via the small-molecule inhibitor ACE-083. Phase II trials demonstrated improvement of the primary end-point (muscle mass of TA and *biceps brachii*), but failure to improve secondary functional outcomes led to abandonment of trials.^57^

A recent trial of p38 inhibitor losmapimod^26^ designed to inhibit DUX4 activity failed to demonstrate improvement of the primary end-point (suppression of four DUX4 target genes) but did demonstrate improvement of secondary functional measures. It would be interesting to measure our FSHD muscle–blood biomarker in blood/muscle samples from patients from this trial. We have also recently developed an *in silico* FSHD muscle fibre for modelling DUX4 dynamics and predicting the consequences of such anti-DUX4 therapies.^64^

In summary, we present the FSHD muscle–blood biomarker, a measure of clinical severity valid in both muscle biopsies and peripheral blood cells. Validation in an independent cohort confirmed our circulating measure as a correlate of disease severity, with particular validity in older patients, raising the possibility of predictive value. The FSHD muscle–blood biomarker represents a powerful tool for routine disease monitoring and for assessing outcomes in clinical trials.^65^

## Supplementary Material

fcad221_Supplementary_Data

## Data Availability

Rahimov *et al*.,^44^ GSE36398, describe 50 muscle biopsies assessed by microarray. Bakay *et al*.,^45^ GSE3307, describe 30 muscle biopsies assessed using microarray. Tasca *et al*.,^46^ GSE26852, describe 15 muscle biopsies assessed by microarray. Osborne *et al*.,^47^ GSE10760, describe 49 muscle biopsies assessed using microarray. Dixit *et al*.,^14^ GSE9397, describe 18 muscle biopsies assessed by microarray. Yao *et al*.,^39^ GSE56787, describe 23 muscle biopsies assessed by RNA-seq (control C6 was removed as it was the only non-quadriceps sample). Wang *et al*.,^21^ GSE115650, describe 43 muscle biopsies assessed by RNA-sequencing. The RNA-sequencing data generated in this study is available upon reasonable request from the European Genome-phenome Archive (https://ega-archive.org) under accession number EGAS00001007350.

## References

[1] Deenen JCW , Arnts H, van der Maarel SM, et al Population-based incidence and prevalence of facioscapulohumeral dystrophy. Neurology. 2014;83(12):1056–1059. 25122204 10.1212/WNL.0000000000000797 PMC4166358

[2] Banerji CRS , Zammit PS. Pathomechanisms and biomarkers in facioscapulohumeral muscular dystrophy: Roles of DUX4 and PAX7. EMBO Mol Med. 2021;13(8):e13695. 34151531 10.15252/emmm.202013695 PMC8350899

[3] Himeda CL , Jones PL. The genetics and epigenetics of facioscapulohumeral muscular dystrophy. Annu Rev Genomics Hum Genet. 2019;20(1):265–291. 31018108 10.1146/annurev-genom-083118-014933

[4] Lemmers RJLF , van der Vliet PJ, Klooster R, et al A unifying genetic model for facioscapulohumeral muscular dystrophy. Science. 2010;329(5999):1650–1653. 20724583 10.1126/science.1189044 PMC4677822

[5] Greco A , Goossens R, Engelen B, Maarel SM. Consequences of epigenetic derepression in facioscapulohumeral muscular dystrophy. Clin Genet. 2020;97(6):799–814. 32086799 10.1111/cge.13726 PMC7318180

[6] Deutekom JCT , Wljmenga C, Tlenhoven EAE, et al FSHD associated DNA rearrangements are due to deletions of integral copies of a 3.2 kb tandemly repeated unit. Hum Mol Genet. 1993;2(12):2037–2042. 8111371 10.1093/hmg/2.12.2037

[7] Wijmenga C , Frants RR, Hewitt JE, et al Molecular genetics of facioscapulohumeral muscular dystrophy. Neuromuscul Disord. 1993;3(5-6):487–491. 8186699 10.1016/0960-8966(93)90102-p

[8] Lemmers RJLF , Tawil R, Petek LM, et al Digenic inheritance of an SMCHD1 mutation and an FSHD-permissive D4Z4 allele causes facioscapulohumeral muscular dystrophy type 2. Nat Genet. 2012;44(12):1370–1374. 23143600 10.1038/ng.2454 PMC3671095

[9] van den Boogaard ML , Lemmers RJLF, Balog J, et al Mutations in DNMT3B modify epigenetic repression of the D4Z4 repeat and the penetrance of facioscapulohumeral dystrophy. Am J Hum Genet. 2016;98(5):1020–1029. 27153398 10.1016/j.ajhg.2016.03.013 PMC4863565

[10] Hamanaka K , Šikrová D, Mitsuhashi S, et al Homozygous nonsense variant in LRIF1 associated with facioscapulohumeral muscular dystrophy. Neurology. 2020;94(23):e2441–e2447. 32467133 10.1212/WNL.0000000000009617 PMC7455367

[11] Hewitt JE , Lyle R, Clark LN, et al Analysis of the tandem repeat locus D4Z4 associated with facioscapulohumeral muscular dystrophy. Hum Mol Genet. 1994;3(8):1287–1295. 7987304 10.1093/hmg/3.8.1287

[12] Gabriëls J , Beckers MC, Ding H, et al Nucleotide sequence of the partially deleted D4Z4 locus in a patient with FSHD identifies a putative gene within each 3.3 kb element. Gene. 1999;236(1):25–32. 10433963 10.1016/s0378-1119(99)00267-x

[13] Kowaljow V , Marcowycz A, Ansseau E, et al The DUX4 gene at the FSHD1A locus encodes a pro-apoptotic protein. Neuromuscul Disord. 2007;17(8):611–623. 17588759 10.1016/j.nmd.2007.04.002

[14] Dixit M , Ansseau E, Tassin A, et al DUX4, a candidate gene of facioscapulohumeral muscular dystrophy, encodes a transcriptional activator of PITX1. Proc Natl Acad Sci U S A. 2007;104(46):18157–18162. 17984056 10.1073/pnas.0708659104 PMC2084313

[15] De Iaco A , Planet E, Coluccio A, Verp S, Duc J, Trono D. DUX-family transcription factors regulate zygotic genome activation in placental mammals. Nat Genet. 2017;49(6):941–945. 28459456 10.1038/ng.3858 PMC5446900

[16] Snider L , Geng LN, Lemmers RJLF, et al Facioscapulohumeral dystrophy: Incomplete suppression of a retrotransposed gene. PLoS Genet. 2010;6(10):e1001181. 21060811 10.1371/journal.pgen.1001181 PMC2965761

[17] Banerji CRS , Zammit PS. PAX7 target gene repression is a superior FSHD biomarker than DUX4 target gene activation, associating with pathological severity and identifying FSHD at the single-cell level. Hum Mol Genet. 2019;28(13):2224–2236. 31067297 10.1093/hmg/ddz043 PMC6586142

[18] Banerji CRS , Panamarova M, Hebaishi H, et al PAX7 target genes are globally repressed in facioscapulohumeral muscular dystrophy skeletal muscle. Nat Commun. 2017;8(1):2152. 29255294 10.1038/s41467-017-01200-4 PMC5735185

[19] Banerji CRS , Panamarova M, Zammit PS. DUX4 expressing immortalized FSHD lymphoblastoid cells express genes elevated in FSHD muscle biopsies, correlating with the early stages of inflammation. Hum Mol Genet. 2020;29(14):2285–2299. 32242220 10.1093/hmg/ddaa053 PMC7424723

[20] van den Heuvel A , Lassche S, Mul K, et al Facioscapulohumeral dystrophy transcriptome signatures correlate with different stages of disease and are marked by different MRI biomarkers. Sci Rep. 2022;12(1):1426. 35082321 10.1038/s41598-022-04817-8 PMC8791933

[21] Wang LH , Friedman SD, Shaw D, et al MRI-informed muscle biopsies correlate MRI with pathology and DUX4 target gene expression in FSHD. Hum Mol Genet. 2019;28(3):476–486. 30312408 10.1093/hmg/ddy364 PMC6337697

[22] Wong CJ , Wang LH, Friedman SD, et al Longitudinal measures of RNA expression and disease activity in FSHD muscle biopsies. Hum Mol Genet. 2020;29(6):1030–1043. 32083293 10.1093/hmg/ddaa031 PMC7158378

[23] Bosnakovski D , Toso EA, Hartweck LM, et al The DUX4 homeodomains mediate inhibition of myogenesis and are functionally exchangeable with the Pax7 homeodomain. J Cell Sci. 2017;130(21):3685–3697. 28935672 10.1242/jcs.205427 PMC5702055

[24] Bosnakovski D , Xu Z, Ji Gang E, et al An isogenetic myoblast expression screen identifies DUX4-mediated FSHD-associated molecular pathologies. EMBO J. 2008;27(20):2766–2779. 18833193 10.1038/emboj.2008.201 PMC2572182

[25] Banerji CRS . PAX7 target gene repression associates with FSHD progression and pathology over 1 year. Hum Mol Genet. 2020;29(13):2124–2133. 32347924 10.1093/hmg/ddaa079

[26] Tawil A , Mellion M, Ronco L, et al Design of a phase 2, randomized, double-blind, placebo-controlled, 24-week, parallel-group study of the efficacy and safety of losmapimod in treating subjects with facioscapulohumeral muscular dystrophy (FSHD): ReDUX4 (1592). Neurology. 2020;94(15 Supplement).

[27] Banerji CRS , Henderson D, Tawil RN, Zammit PS. Skeletal muscle regeneration in facioscapulohumeral muscular dystrophy is correlated with pathological severity. Hum Mol Genet. 2020;29(16):2746–2760. 32744322 10.1093/hmg/ddaa164 PMC7530526

[28] Corasolla Carregari V , Monforte M, Di Maio G, et al Proteomics of muscle microdialysates identifies potential circulating biomarkers in facioscapulohumeral muscular dystrophy. Int J Mol Sci. 2020;22(1):290. 33396627 10.3390/ijms22010290 PMC7795508

[29] Heier CR , Zhang A, Nguyen NY, et al Multi-omics identifies circulating miRNA and protein biomarkers for facioscapulohumeral dystrophy. J Pers Med. 2020;10(4):236. 33228131 10.3390/jpm10040236 PMC7711540

[30] Wong CJ , Wang L, Holers VM, et al Elevated plasma complement components in facioscapulohumeral dystrophy. Hum Mol Genet. 2022;31(11):1821–1829. 34919696 10.1093/hmg/ddab364 PMC9169453

[31] Signorelli M , Mason AG, Mul K, et al Evaluation of blood gene expression levels in facioscapulohumeral muscular dystrophy patients. Sci Reports. 2020;10(1):17547. 10.1038/s41598-020-74687-5 PMC7567883 33067535

[32] Gros M , Nunes AM, Daoudlarian D, et al Identification of serum interleukin 6 levels as a disease severity biomarker in facioscapulohumeral muscular dystrophy. J Neuromuscul Dis. 2022;9(1):83–93. 34459413 10.3233/JND-210711 PMC8842759

[33] Turki A , Hayot M, Carnac G, et al Functional muscle impairment in facioscapulohumeral muscular dystrophy is correlated with oxidative stress and mitochondrial dysfunction. Free Radic Biol Med. 2012;53(5):1068–1079. 22796148 10.1016/j.freeradbiomed.2012.06.041

[34] Statland J , Donlin-Smith CM, Tapscott SJ, van der Maarel S, Tawil R. Multiplex screen of serum biomarkers in facioscapulohumeral muscular dystrophy. J Neuromuscul Dis. 2014;1(2):181–190. 25705588 10.3233/JND-140034 PMC4332410

[35] Ricci G , Ruggiero L, Vercelli L, et al A novel clinical tool to classify facioscapulohumeral muscular dystrophy phenotypes. J Neurol. 2016;263(6):1204–1214. 27126453 10.1007/s00415-016-8123-2 PMC4893383

[36] Lamperti C , Fabbri G, Vercelli L, et al A standardized clinical evaluation of patients affected by facioscapulohumeral muscular dystrophy: The FSHD clinical score. Muscle Nerve. 2010;42(2):213–217. 20544930 10.1002/mus.21671

[37] Choi SH , Gearhart MD, Cui Z, et al DUX4 recruits p300/CBP through its C-terminus and induces global H3K27 acetylation changes. Nucleic Acids Res. 2016;44(11):5161–5173. 26951377 10.1093/nar/gkw141 PMC4914088

[38] Geng LN , Yao Z, Snider L, et al DUX4 activates germline genes, retroelements, and immune mediators: Implications for facioscapulohumeral dystrophy. Dev Cell. 2012;22(1):38–51. 22209328 10.1016/j.devcel.2011.11.013 PMC3264808

[39] Yao Z , Snider L, Balog J, et al DUX4-induced gene expression is the major molecular signature in FSHD skeletal muscle. Hum Mol Genet. 2014;23(20):5342–5352. 24861551 10.1093/hmg/ddu251 PMC4168822

[40] Goeman JJ , Van de Geer S, De Kort F, van Houwelingen HC. A global test for groups of genes: Testing association with a clinical outcome. Bioinformatics. 2004;20(1):93–99. 14693814 10.1093/bioinformatics/btg382

[41] Love MI , Huber W, Anders S. Moderated estimation of fold change and dispersion for RNA-seq data with DESeq2. Genome Biol. 2014;15(12):550. 25516281 10.1186/s13059-014-0550-8 PMC4302049

[42] Robin X , Turck N, Hainard A, et al pROC: An open-source package for R and S+ to analyze and compare ROC curves. BMC Bioinformatics. 2011;12(1):77. 21414208 10.1186/1471-2105-12-77 PMC3068975

[43] Subramanian A , Tamayo P, Mootha VK, et al Gene set enrichment analysis: A knowledge-based approach for interpreting genome-wide expression profiles. Proc Natl Acad Sci U S A. 2005;102(43):15545–15550. 16199517 10.1073/pnas.0506580102 PMC1239896

[44] Rahimov F , King OD, Leung DG, et al Transcriptional profiling in facioscapulohumeral muscular dystrophy to identify candidate biomarkers. Proc Natl Acad Sci U S A. 2012;109(40):16234–16239. 22988124 10.1073/pnas.1209508109 PMC3479603

[45] Bakay M , Wang Z, Melcon G, et al Nuclear envelope dystrophies show a transcriptional fingerprint suggesting disruption of Rb–MyoD pathways in muscle regeneration. Brain. 2006;129(4):996–1013. 16478798 10.1093/brain/awl023

[46] Tasca G , Pescatori M, Monforte M, et al Different molecular signatures in magnetic resonance imaging-staged facioscapulohumeral muscular dystrophy muscles. PLoS One. 2012;7(6):e38779. 22719944 10.1371/journal.pone.0038779 PMC3374833

[47] Osborne RJ , Welle S, Venance SL, Thornton CA, Tawil R. Expression profile of FSHD supports a link between retinal vasculopathy and muscular dystrophy. Neurology. 2007;68(8):569–577. 17151338 10.1212/01.wnl.0000251269.31442.d9

[48] Banerji CRS , Cammish P, Evangelista T, Zammit PS, Straub V, Marini-Bettolo C. Facioscapulohumeral muscular dystrophy 1 patients participating in the UK FSHD registry can be subdivided into 4 patterns of self-reported symptoms. Neuromuscul Disord. 2020;30(4):315–328. 32327287 10.1016/j.nmd.2020.03.001

[49] Young JM , Whiddon JL, Yao Z, et al DUX4 binding to retroelements creates promoters that are active in FSHD muscle and testis. PLoS Genet. 2013;9(11):e1003947. 24278031 10.1371/journal.pgen.1003947 PMC3836709

[50] Jiang S , Williams K, Kong X, et al Single-nucleus RNA-seq identifies divergent populations of FSHD2 myotube nuclei. PLoS Genet. 2020;16(5):e1008754. 32365093 10.1371/journal.pgen.1008754 PMC7224571

[51] Dandapat A , Hartweck LM, Bosnakovski D, Kyba M. Expression of the human FSHD-linked DUX4 gene induces neurogenesis during differentiation of murine embryonic stem cells. Stem Cells Dev. 2013;22(17):2440–2448. 23560660 10.1089/scd.2012.0643 PMC3749711

[52] Banerji CRS , Knopp P, Moyle LA, et al Beta-catenin is central to DUX4-driven network rewiring in facioscapulohumeral muscular dystrophy. J R Soc Interface. 2015;12(102):20140797. 25551153 10.1098/rsif.2014.0797 PMC4277075

[53] Teveroni E , Pellegrino M, Sacconi S, et al Estrogens enhance myoblast differentiation in facioscapulohumeral muscular dystrophy by antagonizing DUX4 activity. J Clin Invest. 2017;127(4):1531–1545. 28263188 10.1172/JCI89401 PMC5373881

[54] Mul K , Horlings CGC, Voermans NC, Schreuder THA, van Engelen BGM. Lifetime endogenous estrogen exposure and disease severity in female patients with facioscapulohumeral muscular dystrophy. Neuromuscul Disord. 2018;28(6):508–511. 29655530 10.1016/j.nmd.2018.02.012

[55] Hangül C , Bozkurt S, Bilge U, et al The ratios of estradiol and progesterone to testosterone influence the severity of facioscapulohumeral muscular dystrophy. Neurol Sci Neurophysiol. 2020;37(4):190.

[56] Knopp P , Krom YD, Banerji CRS, et al DUX4 induces a transcriptome more characteristic of a less-differentiated cell state and inhibits myogenesis. J Cell Sci. 2016;129(20):3816–3831. 27744317 10.1242/jcs.180372 PMC5087662

[57] Kyba M , Bloch RJ, Dumonceaux J, et al Meeting report: The 2020 FSHD international research congress. Skelet Muscle. 2020;10(1):36. 33292505 10.1186/s13395-020-00253-2 PMC7721607

[58] Murdoch B , DelConte C, García-Castro MI. Pax7 lineage contributions to the mammalian neural crest. PLoS One. 2012;7(7):e41089. 22848431 10.1371/journal.pone.0041089 PMC3407174

[59] Blake JA , Ziman MR. Pax genes: Regulators of lineage specification and progenitor cell maintenance. Development. 2014;141(4):737–751. 24496612 10.1242/dev.091785

[60] Das S , Chadwick BP. Influence of repressive histone and DNA methylation upon D4Z4 transcription in non-myogenic cells. PLoS One. 2016;11(7):e0160022. 27467759 10.1371/journal.pone.0160022 PMC4965136

[61] Urbánek P , Wang ZQ, Fetka I, Wagner EF, Busslinger M. Complete block of early B cell differentiation and altered patterning of the posterior midbrain in mice lacking Pax5/BSAP. Cell. 1994;79(5):901–912. 8001127 10.1016/0092-8674(94)90079-5

[62] Heher P , Ganassi M, Weidinger A, et al Interplay between mitochondrial reactive oxygen species, oxidative stress and hypoxic adaptation in facioscapulohumeral muscular dystrophy: Metabolic stress as potential therapeutic target. Redox Biol. 2022;51:102251. 35248827 10.1016/j.redox.2022.102251 PMC8899416

[63] Carnac G , Vernus B, Bonnieu A. Myostatin in the pathophysiology of skeletal muscle. Curr Genomics. 2007;8(7):415–422. 19412331 10.2174/138920207783591672 PMC2647158

[64] Cowley MV , Pruller J, Ganassi M, Zammit PS, Banerji CRS. An in silico FSHD muscle fibre for modelling DUX4 dynamics and predicting the impact of therapy. eLife. 2023;12:e88345. 37184373 10.7554/eLife.88345 PMC10287159

[65] Tihaya MS , Mul K, Balog J, et al Facioscapulohumeral muscular dystrophy: The road to targeted therapies. Nat Rev Neurol. 2023;19(2):91–108. 36627512 10.1038/s41582-022-00762-2 PMC11578282

