# Supplementary material for: The FSHD muscle–blood biomarker: a circulating transcriptomic biomarker for clinical severity in facioscapulohumeral muscular dystrophy
Source: Brain Commun. 2023 Aug 16;5(5):fcad221. doi: 10.1093/braincomms/fcad221 (PMC10507741; doi:10.1093/braincomms/fcad221)
Supplement: fcad221_Supplementary_Data [file fcad221_supplementary_data.zip › Supplementary_Methods.pdf]

The FSHD muscle-blood biomarker: a circulating transcriptomic biomarker for clinical severity in facioscapulohumeral muscular dystrophy

Christopher R. S. Banerji, Anna Greco, Leo A. B. Joosten, Baziel G. M. van Engelen and

Peter S. Zammit

Supplementary Methods

Clinical Assessments

Age, sex, height, weight, BMI, and MRC sum score were recorded. MRC sum score was calculated as the sum of MRC scores from six muscle regions on both sides (upper arm abductors, elbow flexors, wrist extensors, hip flexors, knee extensors and foot dorsal flexors). Total score ranges from 0 (complete paralysis) to 60 (normal muscle strength). Additional clinical features were assessed for FSHD patients including the Ricci score[1], the Lamperti score[2] and maximum voluntary contraction (MVC) of tibialis anterior. Ricci score[1] is a 0-10 severity scale that assumes a descending spread of symptoms from face and shoulders to pelvic and leg muscles typical of FSHD. The Lamperti score[2] comprises a 0-15 severity scale evaluating degree of muscle weakness in 5 muscle regions separately. MVC of tibialis anterior was calculated as described by de Jong et al[3]. Finally, FSHD genetic characteristics (FSHD type and D4Z4 repeat length) and disease duration were obtained (**Supplementary Table 1)**.

PBMCs collection

EDTA blood samples were collected from all participants fulfilling the inclusion criteria at the beginning of each scheduled appointment between 9.00 and 11.00 a.m. PBMCs were isolated from all individuals on the same day and within 2 hours from blood sampling using the protocol described by Gresnigt et al.[4] RNA was extracted from a subset of PBMCs using the commercial RNeasy mini kit Qiagen. Participants were not asked to fast before blood sampling.

PBMCs were isolated by dilution of fresh EDTA blood in sterile phosphate-buffered saline (PBS) and density centrifugation over Ficoll-Paque (GE healthcare, Zeist, The Netherlands) as described[4].

Magnetic resonance imaging

FSHD patients were examined on the same 3T MR system (Tim TRIO, Siemens, Erlangen, Germany) adapting the protocol of Mul et al.[5]. Briefly, Dixon and Turbo Inversion Recovery Magnitude (TIRM) sequences were made using a phased array birdcage coil around upper and lower legs. Dixon sequences quantified the degree of muscle fatty infiltration. Adjustments were made for the DIXON sequence: field of view (FOV) 435 mm, slice thickness 5 mm, gap 5 mm, repetition time (TR) 10 ms, time to echo (TE1 / TE2) 1,26 / 2,49 ms, number of slices per slab 72, flip angle (FA) 3 degree, base resolution 320. For TIRM sequence used to identify inflammation, an inversion time of 240 ms was selected to suppress the fat signal and parameters were: FOV 435 mm, slice thickness 5 mm, gap 10 mm, TR 4140 ms, TE 41 ms, number of slices per slab 28, FA 150 degree, base resolution 320.

Muscle biopsy collection

We collected two paired muscle samples from each of 24 FSHD patients: one MRI-guided muscle biopsy targeting a TIRM negative (non-inflamed) muscle *vastus lateralis* (*vastus intermedius* from one patient) and a second sample from a leg muscle scored as TIRM hyperintense (presumed active inflammation). The area of biopsy was selected on: presence of TIRM hyperintensity, degree of fatty infiltration, amount and location of normal appearing muscle. Muscle biopsy site was marked on the skin with a fish oil marker, positioned on a reference line connecting the anterior superior iliac spine with the tibial tuberosity for upper leg, and the tibial tuberosity with the lateral malleolus for lower leg. Transversal 3D T1-weighted high resolution images (FOV 269 mm, slice thickness 1 mm, TR 759 ms, TE 2,61 ms, number of slices per slab 160, FA 13 degree, base resolution 384), TIRM (FOV 175 mm, slice thickness 4 mm, TR 4100 ms, TE 42 ms, number of slices per slab 23, FA 150 degree, base resolution 256), and DIXON images (FOV 500 mm, slice thickness 5 mm, TR 9,18 ms, TE1 / TE2 1,27 / 2,5 ms, number of slices per slab 52, FA 8 degree, base resolution 384), were made to confirm and determine exact area of biopsy. An experienced interventional radiologist determined needle trajectory and insertion site. Skin was cleaned with chlorhexidine in alcohol or other disinfectant. Biopsy site was infiltrated with 5 ml of 2% lidocaine taking care to inject skin and subcutaneous tissue, but not muscle. A 5 mm incision was made, and the skin layer penetrated with a scalpel blade. A coaxial needle containing a plastic introduction sheath and inner cutting stylet was introduced (ATEC, Hologic, Bedford, USA). The inner needle was then retracted and replaced by a blunt plastic obturator whereafter localizing images confirmed correct needle

position, with trajectory adjusted towards target area as needed. After adjustment, fast verification images were made in at least two planes. The biopsy was taken using a MR compatible 9 gauge vacuum-assisted needle (ATEC, Hologic, Bedford, USA) and a verification image with the plastic obturator in situ confirmed biopsy site and evaluated possible complications. Finally, the sheath was removed and pressure applied over biopsy area to prevent bleeding. Steri-strips and bandage were applied to close the incision.

Bergström needle muscle biopsy from the *vastus lateralis* were collected from 11 control individuals as described[6].

MRI analysis

Dixon MRI sequences were analyzed using MATLAB (version R2020a, The Mathworks, Inc. Natick, Massachusetts, United States) and ImageJ software. A fat fraction (FF) map was calculated using MATLAB from the water and fat image of the Dixon sequence according to:

$$FF = \frac{F}{F + W}$$

Muscle contours of 12 upper leg muscles (*sartorius, gracilis, vastus medialis, vastus lateralis, vastus intermedius, rectus femoris, biceps femoris caput brevis, biceps femoris caput longus, semitendinosus, semimembranosus, adductor magnus, adductor longus*) and 7 lower leg muscles (*tibialis anterior, extensor digitorum longus, peroneus, tibialis posterior, soleus, gastrocnemius medialis, and gastrocnemius lateralis*) muscles were manually outlined on the fat fraction map at specific regions as described[5]. Average fat fraction within one muscle contour ($\overline{FF_i}$) was calculated using ImageJ. Finally, average lower limb fat fraction (LLFF) weighted on the area (A) of each muscle was calculated according to:

$$LLFF = \frac{\sum_{i=1}^{n} A_i \cdot \overline{FF_i}}{\sum_{i=1}^{n} A_i}, n\text{: muscle contours of one patient}$$

We provide the single fat fraction of each muscle (section) and the area of each single muscle alongside weighted average fat fraction and lower limb fat fraction in **Supplementary Table 2**.

Quality control and mapping of RNA-sequencing data

Raw reads were trimmed using trim-galore, utilising cutadapt to remove Illumina Sequencing Adapters at the 3' end. Additionally, 15 bases were trimmed from the 5' end of the reads due to biased distributions. Reads were mapped to the human transcriptome using human genome sequence GRCh38 and v103 gene annotations downloaded from Ensembl, via the Salmon tool (v1.6.0) to correct for fragment GC content bias[7], summarising data at the transcript level. For gene level analysis, read counts assigned to given transcripts were summed over in each sample. The resulting matrix of read counts was analysed using R.

Biomarker comparison across FSHD and control samples via multivariate regression and Globaltest

Biomarker values in control samples (PBMCs, muscle biopsies) were compared with corresponding FSHD samples (PBMCs, TIRM- and TIRM+ muscle biopsies), separately for each tissue type, via multivariate linear regression. Each biomarker was modelled as a function of age, sex and a disease variable encoding FSHD or TIRM status. In the case of PBMCs the disease variable took binary values: 0 denoting control and 1 denoting FSHD. In the case of muscle biopsies the disease variable took categorical values: 'control', 'TIRM-' or 'TIRM+'. Biomarker values in isogenic TIRM+ and TIRM- muscle biopsies were modelled via a separate multivariate analysis as a function of patient sampled and the disease variable. Significance was assessed at $p<0.05$.

Globaltest was implemented in R via the corresponding Bioconductor package[8], analogous to the above multivariate analysis using 2 separate analyses. In the first the response vector was set as the above disease variable and dependent variables were age and sex and genes comprising each biomarker. In the second analysis limited to FSHD samples the response vector described TIRM+ and TIRM- samples and dependent variables were age, sex, patient sampled and genes comprising each biomarker. In both scenarios globaltest was implemented for each biomarker, employing genes comprising the PAX7 score, the 3 sets of DUX4 target genes and the Lymphoblast score as covariates. Significance was assessed at $p<0.05$. (**Supplementary Table 3**).

Datasets employed in meta-analysis of FSHD muscle-blood biomarker

Rahimov et al.,[9] GSE36398, describes 50 muscle biopsies assessed by microarray. Bakay et al.,[10] GSE3307, describes 30 muscle biopsies assessed by microarray. Tasca et al.,[11] GSE26852, describes 15 muscle biopsies assessed by microarray. Osborne et al.,[12] GSE10760, describes 49 muscle biopsies assessed by microarray. Dixit et al.,[13] GSE9397, describes 18 muscle biopsies assessed by microarray. Yao et al.,[14] GSE56787, describes 23 muscle biopsies assessed by RNA-seq (control C6 was removed as it was the only non-quadriceps sample). Wang et al.,[15] GSE115650, describes 43 muscle biopsies assessed by RNA-sequencing.

References

1. Ricci, G. *et al.* A novel clinical tool to classify facioscapulohumeral muscular dystrophy phenotypes. *J. Neurol.* **263,** 1204–1214 (2016).

2. Lamperti, C. *et al.* A standardized clinical evaluation of patients affected by facioscapulohumeral muscular dystrophy: The FSHD clinical score. *Muscle and Nerve* **42,** 213–217 (2010).

3. de Jong, L. *et al.* Three-dimensional quantitative muscle ultrasound in a healthy population. *Muscle Nerve* **64,** 199–205 (2021).

4. Gresnigt, M. S. *et al.* Aspergillus fumigatus–Induced IL-22 Is Not Restricted to a Specific Th Cell Subset and Is Dependent on Complement Receptor 3. *J. Immunol.* **190,** 5629–5639 (2013).

5. Mul, K. *et al.* Adding quantitative muscle MRI to the FSHD clinical trial toolbox. *Neurology* **89,** 2057–2065 (2017).

6. Tarnopolsky, M. A., Pearce, E., Smith, K. & Lach, B. Suction-modified Bergström muscle biopsy technique: experience with 13,500 procedures. *Muscle Nerve* **43,** 716–725 (2011).

7. Patro, R., Duggal, G., Love, M. I., Irizarry, R. A. & Kingsford, C. Salmon: fast and bias-aware quantification of transcript expression using dual-phase inference. *Nat. Methods* **14,** 417 (2017).

8. Goeman, J. J., Van de Geer, S., De Kort, F. & van Houwellingen, H. C. A global test for groups of genes: testing association with a clinical outcome. *Bioinformatics* **20,** 93–99 (2004).

9. Rahimov, F. *et al.* Transcriptional profiling in facioscapulohumeral muscular dystrophy to identify candidate biomarkers. *Proc. Natl. Acad. Sci. U. S. A.* **109,** 16234–9 (2012).

10. Bakay, M. *et al.* Nuclear envelope dystrophies show a transcriptional fingerprint suggesting disruption of Rb–MyoD pathways in muscle regeneration. *Brain* **129,** 996–1013 (2006).

11. Tasca, G. *et al.* Different Molecular Signatures in Magnetic Resonance Imaging-Staged Facioscapulohumeral Muscular Dystrophy Muscles. *PLoS One* **7,** e38779 (2012).

12. Osborne, R. J., Welle, S., Venance, S. L., Thornton, C. A. & Tawil, R. Expression profile of

FSHD supports a link between retinal vasculopathy and muscular dystrophy. *Neurology* **68,** 569–577 (2007).

13. Dixit, M. *et al.* DUX4, a candidate gene of facioscapulohumeral muscular dystrophy, encodes a transcriptional activator of PITX1. *Proc. Natl. Acad. Sci.* **104,** 18157–18162 (2007).

14. Yao, Z. *et al.* DUX4-induced gene expression is the major molecular signature in FSHD skeletal muscle. *Hum. Mol. Genet.* **23,** 5342–52 (2014).

15. Wang, L. H. *et al.* MRI-informed muscle biopsies correlate MRI with pathology and DUX4 target gene expression in FSHD. *Hum. Mol. Genet.* **28**, 476-486 (2019).
