# Supplementary material for: The FSHD muscle–blood biomarker: a circulating transcriptomic biomarker for clinical severity in facioscapulohumeral muscular dystrophy
Source: Brain Commun. 2023 Aug 16;5(5):fcad221. doi: 10.1093/braincomms/fcad221 (PMC10507741; doi:10.1093/braincomms/fcad221)
Supplement: fcad221_Supplementary_Data [file fcad221_supplementary_data.zip › Banerji et al doi. 10.1093braincommsfcad221 Supplementary Figure.pdf]

The FSHD muscle-blood biomarker: a circulating transcriptomic biomarker for clinical severity in facioscapulohumeral muscular dystrophy

Christopher R. S. Banerji, Anna Greco, Leo A. B. Joosten, Baziel G. M. van Engelen and Peter S. Zammit

Supplementary Figure

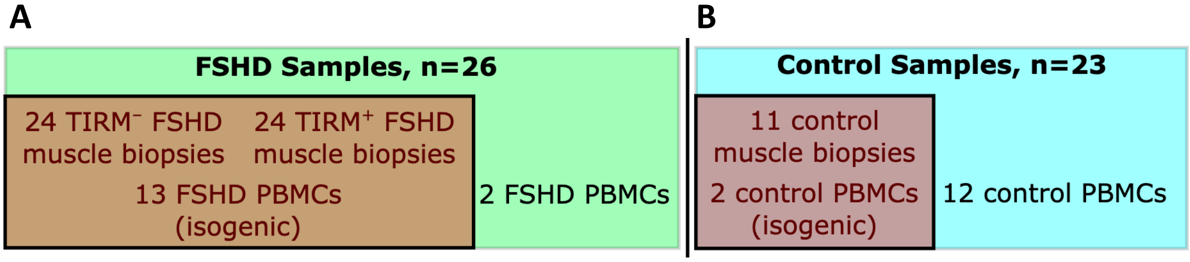

Supplementary Figure 1: Overview of patient cohort and samples obtained

(A, B) Summary of the numbers of (A) TIRM$^-$ and TIRM$^+$ muscle biopsies and blood samples obtained from each FSHD patient and (B) each control individual, used for RNA-sequencing and other analysis.
